# A Scoping Review on the Influence of Housing on the Health and Well-Being of People with a Spinal Cord Injury/Dysfunction

**DOI:** 10.3390/healthcare12242537

**Published:** 2024-12-16

**Authors:** Sarmitha Sivakumaran, Tsione Kebede, Kirstin E. Yuzwa, Ella C. N. Wong, Christine L. Sheppard, Sara J. T. Guilcher, Peter Athanasopoulos, Krista L. Best, Anita Kaiser, Vanessa K. Noonan, Sander L. Hitzig

**Affiliations:** 1 Rehabilitation Sciences Institute, Temerty Faculty of Medicine, University of Toronto, Toronto, ON M5G 1V7, Canada; sarmitha.sivakumaran@mail.utoronto.ca (S.S.); sara.guilcher@utoronto.ca (S.J.T.G.); anita.kaiser@uhn.ca (A.K.); 2 St. John’s Rehab Research Program, Sunnybrook Research Institute, Sunnybrook Health Sciences Centre, North York, ON M2M 2G1, Canada; kebedet@my.yorku.ca (T.K.); wonge72@mcmaster.ca (E.C.N.W.); 3 Faculty of Science, York University, North York, ON M3J 1P3, Canada; 4 Faculty of Science, Department of Kinesiology, McMaster University, Hamilton, ON L8S 4L8, Canada; 5 Wellesley Institute, Toronto, ON M5A 2E7, Canada; christine.sheppard@wellesleyinstitute.com; 6 Factor-Inwentash Faculty of Social Work, University of Toronto, Toronto, ON M5S 1V4, Canada; 7 Leslie Dan Faculty of Pharmacy, University of Toronto, Toronto, ON M5S 3M2, Canada; 8 Department of Physical Therapy, Temerty Faculty of Medicine, University of Toronto, Toronto, ON M5G 1V7, Canada; 9 Spinal Cord Injury Ontario, Toronto, ON M4G 3V9, Canada; 10 School of Rehabilitation Sciences, Faculté de médecine, Université Laval, Québec City, QC G1V 0A6, Canada; krista.best@fmed.ulaval.ca; 11 Centre Interdisciplinaire de Recherche en Réadaptation et Intégration Sociale (CIRRIS), Québec City, QC G1M 2S8, Canada; 12 KITE Research Institute, University Health Network, Toronto, ON M5G 2A2, Canada; 13 Praxis Spinal Cord Institute, Vancouver, BC V5Z 1M9, Canada; vnoonan@praxisinstitute.org; 14 Department of Occupational Science and Occupational Therapy, Temerty Faculty of Medicine, University of Toronto, Toronto, ON M5G 1V7, Canada; 15 Dalla Lana School of Public Health, University of Toronto, Toronto, ON M5T 3M7, Canada

**Keywords:** spinal cord injuries, housing, health, quality of life

## Abstract

Background/Objectives: Despite the growing recognition of housing as a significant concern for individuals with a spinal cord injury/dysfunction (SCI/D), there is limited research available on this topic. This scoping review aimed to identify and describe the literature on housing across the continuum for people with an SCI/D. Methods: This review utilized Arksey and O’Malley’s scoping review framework. Five databases were searched including MEDLINE (Ovid), Embase (Ovid), CINAHL Plus (EBSCO), PsycINFO (Ovid), and Web of Science (Core Collection). In addition, Google’s Advanced Search function was used to search the gray literature, and reference lists from the included studies were scanned. A preliminary assessment of the Theory of Access (TOA) constructs and their relationships was conducted using Penchansky and Thomas’ Theory of Access and Saurman’s additional updates. An adapted version of this theory was developed by the research team to identify the types of studies that assess the TOA’s six constructs and was used to identify knowledge gaps to advance research in this field. Results: The search yielded 25,861 records, with 36 studies meeting the inclusion criteria. Data analysis revealed the participants’ sociodemographic and impairment characteristics, as well as essential information pertaining to housing across the continuum for individuals with an SCI/D, both of which were inconsistently reported across the studies. Several studies (*n* = 18) reported on the influence of home adaptations on the health and well-being of individuals with an SCI/D. When framed within the TOA, issues of accessibility presented the most substantial barriers for the SCI/D community, followed by acceptability and availability. Conclusions: The findings of this scoping review suggest that housing is an understudied topic and that further research is required to generate evidence to better support the housing needs of individuals with an SCI/D globally.

## 1. Introduction

A spinal cord injury/dysfunction (SCI/D) is a significant, life-changing event, which is linked to a traumatic event (e.g., an accident) or a non-traumatic event (e.g., a tumor on the spine, degeneration) [1]. Following an SCI/D, individuals may experience paralysis, changes in sensation, and autonomic dysfunction, such as challenges with their bladder and bowel control [1]. An SCI/D is also associated with a host of chronic or episodic secondary health conditions, including but not limited to pressure injuries, musculoskeletal and/or neuropathic pain, and urinary tract infections [2]. In addition to the negative impacts on overall health and quality of life (QoL), the physical, mental, and social impairments and health conditions associated with an SCI/D have significant implications for housing due to the changes in the individuals’ functioning and health, particularly concerning their emerging needs for accessibility.

According to the Canadian Human Rights Commission, accessible housing refers to “housing that is designed and built to meet specific accessibility needs” [3]. Living in an inaccessible home has consequences for the health and well-being of people with accessibility needs [4]. Access to appropriate and affordable housing is a human right and is critical for promoting health, safety, dignity, inclusion, and community participation [5]. Individuals with disabilities encounter challenges in communicating with housing providers, obtaining information about available and accessible housing units, and having requests for home modifications approved or responded to adequately, which prevents them from making informed decisions about housing [6]. 

Following an SCI/D, most individuals must learn how to re-adapt and navigate their surrounding environments, with the available evidence on housing post-SCI/D indicating that it is a significant issue influencing the SCI/D population’s independence and well-being [7]. A lack of accessible housing among the SCI/D community presents challenges in utilizing their physical spaces, contributes to reduced feelings of safety [8], negatively impacts their dignity and independence [9,10], and leads to an increased reliance on caregiving support [10]. For instance, one study examining the unmet needs of Canadians with an SCI/D (N = 1,549) found that 82.4% of SCI/D participants had accessible housing needs and reported receiving help from government agencies, friends and family, and community organizations, with community organizations providing peer support for 77% who expressed a need for help with their housing [11]. There is evidence that home modifications (e.g., the inclusion of a ramp [10], an accessible bathroom [10,12], etc.) are critical for an improved QoL for this population [12]. When home modifications are enacted, it leads to improved autonomy [12]. 

There are several factors influencing housing outcomes post-SCI/D, such as the level and severity of the injury [13]. For instance, one report [13] revealed that 88% of participants with an American Spinal Injury Association (ASIA) Impairment Scale (AIS) [14] grade of A (AIS-A) to C (AIS-C) had a higher need for accessible housing compared to 33% of AIS-D participants. Hence, those with greater levels of motor or sensory impairment will likely face more difficulties in finding suitable home environments or navigating their homes if not well-adapted to their functional capabilities. 

In addition to impairment, finances are a critical component of being able to obtain accessible housing. The financial constraints caused by restricted sources of income and the higher costs of care post-SCI/D [15,16] may impact the ability to move to a new home or to renovate an existing home to be more accessible. The high unemployment rates among the SCI/D population [17] further restrict the necessary finances to acquire suitable and accessible housing. In Canada, 62.3% (*n* = 824) were employed prior to their injury, whereas only 32.3% (*n* = 426) were employed following their injury [17]. Further compounding the challenges of securing housing is the lack of available and affordable stock [18]. Despite the growing evidence on the impact of inaccessible housing on the health and well-being of individuals with disabilities, it is a relatively under-studied issue [19] and particularly so for the SCI/D population. As a result, further work is required to fully understand the influence of housing (e.g., housing type, residence characteristics, etc.) on this population’s outcomes.

To advance our understanding of housing across the continuum following an SCI/D, a scoping review was undertaken to map the relevant literature to describe what is known about the topic and to identify knowledge gaps in this field. This process can help to advance research in this domain, which can generate the necessary evidence to improve housing outcomes for the SCI/D community.

## 2. Methods

A scoping review was conducted, given our objective of mapping the available literature on an SCI/D and housing across the continuum. Unlike systematic reviews, which aim to evaluate the quality of evidence in a particular field, the goals of scoping reviews are to explore the breadth and depth of evidence within a specific field, to identify knowledge gaps, and to clarify concepts [20,21]. This review was guided by Arksey and O’Malley’s scoping review framework [20], with updates outlined by Levac et al. [22]. To ensure rigor in the scoping review methodology, we followed the Preferred Reporting Items for Systematic Reviews and Meta-Analyses (PRISMA) Extension for Scoping Reviews (PRISMA-ScR) as outlined by Tricco et al. [23]. 

Arksey and O’Malley’s scoping review framework [20], with the recommendations of Levac et al. [22], includes the following: (i) identifying the research question; (ii) identifying relevant studies; (iii) study selection; (iv) charting the data, (v) collating, summarizing, and reporting the results; and (vi) a consultation exercise [20,22]. The optional sixth step, a consultation exercise [20,22], was not undertaken for this scoping review. Consistent with the scoping review methodology, a risk of bias assessment of the included studies was not conducted [21]. The scoping review protocol was registered on the Open Science Framework (OSF) Registry at https://osf.io/4fj78/ (accessed on 16 August 2024).

### 2.1. Guiding Framework

Penchansky and Thomas’ Theory of Access (TOA) [24] was used as a guiding theoretical framework, including the addition of one construct by Saurman [25]. The TOA comprises six constructs of access, including availability, accessibility, adequacy/accommodation, affordability, acceptability, and awareness (see Appendix A) [24,25]. This theory aims to better understand how access influences individuals’ decisions in obtaining the most essential health services required for their daily activities [24,25]. With regards to housing, there are several applications of the TOA that are well suited for the present scoping review. First, the TOA offers the potential to explore the interactions between an individual’s physical capacity and their surrounding environment, and it has been recommended for exploring housing for people with mobility impairments, such as an SCI/D [26]. Second, Barrow and Pollack assert that the interconnected nature of the TOA constructs can support the examination of the potential relationships between governmental policies, individuals seeking housing, and landlords [27]. Furthermore, the application of Penchansky and Thomas’ framework has been previously suggested to explore equity within the realms of housing and health [27]. Hence, it was determined that the TOA could facilitate insights on critical aspects of housing addressed in the SCI/D literature to date, while uncovering potential knowledge gaps.

To gain a better understanding of how the literature addresses the six constructs, a preliminary assessment of the included studies was conducted to categorize which TOA construct(s) the literature is classified under. An adapted version of the TOA was created and iteratively revised by members of the research team with expertise in accessible housing, an SCI/D, and occupational therapy (S.S., K.E.Y., and S.L.H.) (See Table 1).

### 2.2. Identifying the Research Question

We used the population, context, concept (PCC) framework [28] to inform our research question. The population included individuals with an SCI/D, and housing was the concept. We deliberately considered all contexts, without restricting the geographical location, as we aimed to gather all information related to housing for the SCI/D population across the continuum. Furthermore, this included examining studies that provided details on housing type, living situation, residence characteristics, home modifications, and the perceived acceptability or suitability of homes, among other aspects. Therefore, this scoping review addressed the following research question: *What is the current state of evidence about housing across the continuum for individuals with an SCI/D?* Overall, a broad lens was applied to explore various domains of housing, which were sensitized via the TOA. Furthermore, this scoping review addressed the following objectives: (i) to map the current state of evidence concerning an SCI/D and housing (e.g., housing type, residence characteristics, living situation, etc.), (ii) to describe the influence of (in)accessible housing on well-being, and (iii) to conduct a preliminary assessment of the included studies using an adapted version of the TOA.

### 2.3. Identifying Relevant Studies

A search strategy was developed in consultation with a health sciences librarian from the University of Toronto (see Appendix B). A few key articles were identified by members of the research team (S.S. and S.L.H.) to pilot the search strategy. Five databases were searched from the database inception until August 27, 2024, including MEDLINE (Ovid), Embase (Ovid), CINAHL Plus (EBSCO), PsycINFO (Ovid), and Web of Science (Core Collection). Furthermore, terms from the database search, including but not limited to “spinal cord injury”, “tetraplegia”, “paraplegia”, “housing”, and “home”, were also used to search the gray literature using Google’s Advanced Search function. The gray literature needed sufficient detail on an SCI/D and housing, including but not limited to housing across the continuum, the influence of housing on health and well-being, discussions about housing preferences, or features of the built environment, to be considered for inclusion. Finally, reference lists from the included studies were scanned to identify additional studies. 

### 2.4. Study Selection

The selected records were screened against the following inclusion criteria: (i) published in 2010 or later to ensure the identified literature was representative of current housing trends and policies; (ii) published in English; and (iii) focused on housing for individuals with an adult onset of an SCI/D. No restrictions were placed on geographic location. The exclusion criteria included poster presentations, literature reviews, published abstracts, conference proceedings, case reports, and case series. Covidence [29], a data screening and extraction tool, was used to screen all the identified records. Two reviewers (S.S., T.K., or E.C.N.W.) independently reviewed all records during the title and abstract screening and full-text review. Conflicts were resolved through discussion between two reviewers (S.S., T.K., or E.C.N.W.), with an expert member of the research team (S.L.H.) assisting in conflict resolution as needed. Before excluding studies due to the unavailability of full texts, attempts were made to contact researchers where possible.

### 2.5. Charting the Data

A data extraction table was developed and iteratively revised by the research team (Appendix C). The information extracted from the studies included the following: (i) authors, (ii) year of publication, (iii) country of study origin, (iv) aim(s) of study, (v) study design, (vi) outcome measures, (vii) sample size, (viii) population studied, (ix) level and severity of injury, (x) duration and/or range of injury, (xi) mean age and/or age range of participants, (xii) sex and/or gender of participants, (xiii) race and/or ethnicity of participants, (xiv) household income, (xv) employment status, (xvi) housing type, (xvii) living situation, (xviii) residence characteristics (e.g., home ownership, geographical location of home, etc.), (xix) key findings and/or conclusions related to housing, and (xx) TOA construct(s). To pilot the data extraction table, two reviewers (S.S., T.K., or E.C.N.W.) independently extracted the data from three key articles chosen from all studies deemed eligible for inclusion, using Microsoft Excel. The reviewers then met with an expert member of the research team (S.L.H.) to discuss discrepancies and revise the data extraction table as required. Subsequently, the reviewers independently extracted all the identified records using Covidence and ensured a consensus. Discrepancies were resolved through discussion between the reviewers, with a third reviewer (S.L.H.) assisting in conflict resolution as required. 

### 2.6. Collating, Summarizing, and Reporting the Results

The results in subsequent sections were collated and presented in accordance with the reporting guidelines outlined by Tricco et al. [23]. The characteristics of the included studies and findings related to housing across the continuum for individuals with an SCI/D were summarized.

## 3. Results

**Figure 1:**
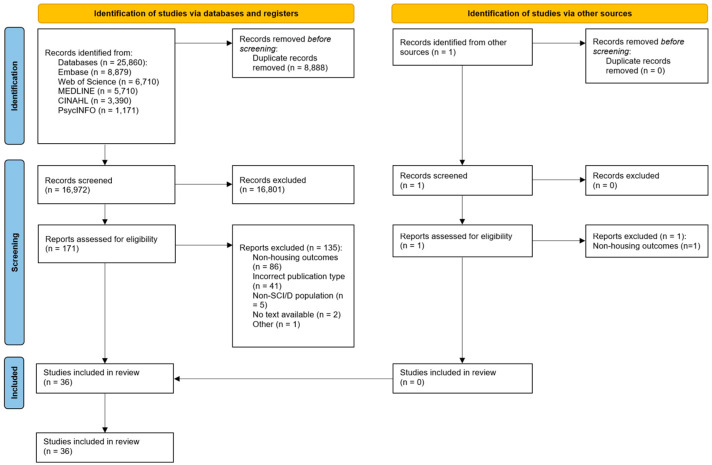
PRISMA flow diagram.

Figure 1 presents a PRISMA diagram that summarizes the screening process. The initial search yielded 25,861 records. After de-duplication, 16,973 studies were screened, and 172 full-text studies were assessed for eligibility. This review included 34 peer-reviewed articles [7,8,9,10,11,12,30,31,32,33,34,35,36,37,38,39,40,41,42,43,44,45,46,47,48,49,50,51,52,53,54,55,56,57] and two gray literature sources [58,59].

### 3.1. Study Characteristics

Table S1 (Supplementary Materials) summarizes the 36 studies. The thirty-six included studies employed the following study designs: fifteen employed a cross-sectional design [9,11,38,41,42,45,48,49,51,52,53,54,57,58,59], nine employed a qualitative design [7,8,12,34,36,44,46,50,56], three were cohort studies [35,40,55], two used a mixed-methods design [33,39], one was a descriptive study [10], one used an adopted mixed-methods qualitative and quantitative approach [37], one used a retrospective analysis [31], one employed a longitudinal prospective design [32], one was an experimental study [47], one was a community-based randomized controlled trial (RCT) [30], and one study employed Q methodology [43]. Q methodology explores individuals’ subjective viewpoints about a topic by asking them to rank statements according to their personal perspectives [60].

The studies were conducted in the following regions: twelve in North America (with six in Canada [11,33,39,41,43,44] and six in the United States of America (USA) [9,31,36,37,50,57]); fourteen in Europe (five in the United Kingdom (UK) [8,35,40,46,56], three in Sweden [48,49,52], two in Switzerland [38,53], one in Spain [58], one in Italy [47], one in France [51], and one in Finland [54]); five in Asia (one in China [32], one in Mongolia [34], one in Pakistan [30], one in Nepal [55], and one in Iran [12]); two in Africa (in South Africa [45,59]); two in Australia [7,42]; and one in South America (in Brazil [10]).

### 3.2. TOA Constructs and Outcome Measures

An adapted version of the TOA (Table 1), developed by the research team (S.S., K.E.Y., and S.L.H.), was used to assess the 36 included articles. The frequency counts for the six TOA constructs are presented in Table S1 and are as follows: accessibility (*n* = 33), acceptability (*n* = 32), availability (*n* = 25), adequacy/accommodation (*n* = 24), affordability (*n* = 14), and awareness (*n* = 13).

In terms of housing- or environment-related outcome measures, one study used the Nottwil Environmental Factors Inventory (NEFI) [45], two used the NEFI Short Form (NEFI-SF) [42,54], three used the Housing Enabler Tool [48,49,52], and one used the Craig Hospital Inventory of Environmental Factors Short Form (CHIEF-SF) [40]. The NEFI is a questionnaire that assesses how environmental factors impact participation (e.g., productive life, social life, and community life) [61]. The Craig Hospital Inventory of Environmental Factors (CHIEF) evaluates how often and how significantly perceived barriers (e.g., physical, attitudinal, and policy) prevent individuals with disabilities from pursuing their desired activities or needs [62]. The Housing Enabler measures the fit between a person and the physical accessibility of their home by using a weighted scale for home environment features based on the relative accessibility impact for individuals with specific functional impairments and mobility devices [63]. Other measures used across the studies included, but were not limited to, the Quality of Life Questionnaire (QoLQ), Functional Independence Measure (FIM), Life Satisfaction Questionnaire, 12-Item Short Form Survey (SF-12), and Spinal Cord Independence Measure (Table S1).

### 3.3. SCI/D Populations

The majority of studies (*n* = 28) focused exclusively on individuals with an SCI/D [8,9,10,11,12,30,31,32,33,34,35,36,38,39,40,42,45,47,48,49,50,52,53,54,55,56,57,58]. Four studies included participants with an SCI/D and an additional group with another condition (e.g., traumatic brain injury, multiple sclerosis, etc.) [37,41,51,59]. Three studies involved individuals with an SCI/D or another condition, as well as their household family members [7,43,44], and one study included participants with metastatic spinal cord compression [46].

### 3.4. Sociodemographic and Impairment Characteristics

All studies included some sociodemographic and impairment characteristics; however, there were discrepancies in the reporting of these characteristics across the 36 studies (see into Supplementary Materials for full details). The participants’ ages across the studies ranged from 16 to 94 years. In majority of the studies (*n* = 29), the participant cohort was comprised of more males than females. Three studies did not report the sex or gender of their participants [35,40,53]. One study comprised an equal number of male and female participants [7]. Three studies comprised more female participants [41,43,44]. Eight studies provided information on participant ethnicity, including non-Hispanic White, non-Hispanic Black, Hispanic, Caucasian, African American, White, African American, Pacific Islander, or other equity-deserving groups [9,11,31,37,42,46,50,57]. Additionally, nine studies reported the household income of participants, either monthly or annually, or the range [9,11,31,33,39,42,45,57,58]. Nineteen studies reported the employment status of the participants or how their income was acquired (e.g., work, disability pension, etc.) [8,9,10,11,31,32,34,36,37,39,40,42,43,44,50,51,54,55,57]. Of the nineteen studies, several further revealed that more individuals with an SCI/D were unemployed compared to those who were employed [9,10,11,31,32,34,37,39,43,44,50,54,55,57]. The majority of the studies reported either the level, the severity, or both the level and severity of the SCI/D among the participants. Some studies also reported the level and type of injury based on the ASIA AIS Scale [14]. 

### 3.5. Housing Type, Living Situation, and Residence Characteristics

The participants’ housing type, living situation, and residence characteristics were reported inconsistently across the 36 studies. Eleven studies [9,10,32,33,35,44,48,49,50,51,56] reported housing type (e.g., house, apartment, condominium, etc.), eighteen studies [7,9,10,11,33,37,38,39,42,43,44,48,49,50,51,52,54,55] reported living situation (e.g., living with someone or living alone), and sixteen studies [9,10,11,31,33,34,38,39,42,43,44,48,49,55,57,58] reported residence characteristics (e.g., residing in urban or rural areas, home ownership, etc.).

Among the studies that reported housing type, Botticello and colleagues found that 73.3% (N = 690) of participants with an SCI/D lived in a house or condominium compared to other types of dwellings [9]. Similarly, Caro and Cruz reported that most participants lived in houses, with one residing in an apartment and one residing on a farm [10]. Norin and colleagues offered detailed data on housing situations based on different AIS scales [48]. For participants with tetraplegia (AIS A-C), the study by Norin et al. revealed that 50% resided in multi-dwelling blocks and 50% in single-family houses [48]. Participants with paraplegia (AIS A-C) had a slightly different distribution, with 46% residing in multi-dwelling blocks and 54% in single-family houses [48]. Lastly, among participants with AIS-D, 42% lived in multi-dwelling blocks, while 58% resided in single-family houses [48].

Among the studies that reported the living situation, 14 studies indicated that more participants lived with others rather than alone [7,9,10,11,33,37,38,39,42,43,44,50,54,55]. For instance, Palimaru and colleagues detailed that one participant resided in a residential home, four lived alone without caregiver support, eight lived with family members who provided live-in caregiver support, one lived with non-family live-in caregivers, six resided with someone who was not a caregiver, and twelve resided with their partner at time of interview [50]. In contrast, Norin et al. [49] and Pettersson et al. [52] found that slightly more participants lived alone compared to those living with others.

Six studies addressed home ownership [9,32,33,43,44,55]. Labbé and colleagues reported that 25 of the 29 participants with an SCI/D owned their homes, as did 26 of the 30 household family members [43]. Participants had lived in their homes for periods ranging from 6 months to 32 years [43]. Most participants resided in households of two (41.38% of those with an SCI/D and 40.00% of household members) or three (41.38% of those with an SCI/D and 40.00% of household members) [43]. In a different study, Scovil et al. found that nineteen participants owned their homes, while five participants rented their homes [55].

### 3.6. Home Modifications and Home Automation

Eighteen studies reported on the impact or perceived impact of home modifications or adaptations for individuals with an SCI/D [7,10,12,30,33,36,38,39,41,43,44,47,49,50,52,55,57,58]. Common home adaptations included modifications to bathrooms and doorways, as well as the inclusion of ramps [36]. A cross-sectional study by Norin et al. found that, although many homes included adaptations such as ramps and adjustable cupboards, many individuals still encountered environmental challenges including uneven levels and difficulty accessing specific items or controls [49]. Moreover, lower secondary health conditions were linked to improved access to home amenities and appliances, reflecting better health outcomes, as revealed by a cross-sectional study [57].

Of the eighteen studies that examined the impact of home modifications, two specifically focused on home automation for individuals with an SCI/D [7,47]. In their phenomenological study, Cleland et al. identified two major categories: (1) the positive outcomes of home automation systems, which facilitated greater independence, and (2) the difficulties related to prolonged assessment and installation processes concerning home automation [7]. The types of home automation installed by participants included lights, switches, door intercoms, automatic doors, heating and cooling systems, blinds, curtains, fans, media controls, taps, showers, and smoke alarms [7]. Similarly, the experimental study by Maresca et al. found that home automation training significantly improved participants’ independence, personal development, functional abilities, social engagement, activities of daily living, and QoL [47].

With regard to environmental challenges, Noreau and colleagues reported that the overall process of meeting needs, as well as the associated costs, significantly impacted the fulfillment of those needs, such as income support and attendant care, with accessible housing facing the most substantial barriers overall [11]. 

Khalili and colleagues reported in their cross-sectional study that wheeled mobility assistive device (WMAD) users (SCI/D, *n* = 76) consistently reported that home modifications have a substantial effect on their autonomy [41]. Common barriers within the home environment included problems with doorways, level variations, reach difficulties, and stairs [41]. The participants expressed more challenges with navigating uneven surfaces (e.g., curbs, stairs), rough terrain (e.g., sand, snow, and unpaved surfaces), and variable environmental factors in outdoor environments, which they found more difficult than those within the home or other buildings [41].

In terms of accessibility outside the home, Caro and Cruz reported that around 90.90% (*n* = 10) of participants made changes to both their homes and sidewalks, with the most common adjustment being the installation of lowered ramps or guides (*n* = 8) [10]. Common barriers identified included windows or doors (*n* = 10), unlevel floors (*n* = 6), and gaps (e.g., holes) (*n* = 4) within the home; unlevel floors (*n* = 11), steep ramps (*n* = 4), and inclined floors (*n* = 4) on the sidewalk; and similar obstacles in the surrounding block in which participants’ homes were located [10].

A cross-sectional study by Angulo et al. explored the use of home adaptations among individuals with an SCI/D (N = 105) and found that home modifications enhanced their independence and QoL [58]. This finding is further supported by another study, in which one participant with tetraplegia noted a reduction in feelings of worry because of their home modifications [7]. Angulo et al. further noted that, while 81.9% of individuals with an SCI/D had their homes modified, 18.1% had not, reflecting the barriers posed by financial constraints, time limitations, or the necessity of relocating to a home with appropriate modifications [58]. Finally, a significant association was reported between QoLQ scores and adapted housing among this population [58].

### 3.7. Influence of Severity of Injury on Housing

One cross-sectional study examined the home environment and participation levels of older adults with a chronic SCI/D, highlighting the impact of injury severity on their living conditions [48]. The study also assessed how specific, measurable housing features influenced the ability of individuals with a chronic SCI/D to participate in various activities [48]. The results indicated that the All AIS D group made fewer home modifications, with only 60% of participants implementing changes, in contrast to over 90% in the Paraplegia AIS A-C group and Tetraplegia AIS A-C group [48]. The All AIS D group encountered more environmental challenges, especially in indoor settings, compared to both the Paraplegia AIS A-C group and Tetraplegia AIS A-C group [48]. Compared to the Paraplegia AIS A-C and All AIS D groups, the Tetraplegia AIS A-C group faced more accessibility challenges (e.g., related to housing), particularly in both indoor and outdoor spaces [48]. Norin et al. reported that older adults with a chronic SCI/D, who encounter significant accessibility barriers tend to experience decreased levels of participation and face greater challenges in engaging in activities [48]. A longitudinal prospective design by Chan and Chan provided insights into the types of assistive equipment used by AIS D participants, including relief seat cushions and commode/shower chairs [32].

### 3.8. Moving After an SCI/D

Two studies investigated residential relocation following an SCI/D [9,31], while two studies [33,53] explored this issue to a limited extent. The mixed-methods study conducted by Coulombe and colleagues found that 48.48% of participants with an SCI/D had relocated since the time of their injury [33]. Some participants cited negative factors within their home, such as its size and family dynamics, as reasons for the likelihood of their relocation [33]. Conversely, others reported that the well-being of their family members made them less inclined to move [33]. Ronca and colleagues revealed that 60% of the 492 participants who completed an aging-related questionnaire expressed interest in relocating to housing that would better accommodate their needs as they age [53]. Relocating closer to an SCI/D center as they aged was reported among 28% of the 492 respondents [53]. According to the cross-sectional study by Botticello and colleagues, the primary reasons for moving within the past year included obtaining a home with improved accessibility (45.1%), improved housing quality (38.9%), accessing places with improved accessibility (28.3%), and wanting an independent household (27.4%) [9]. Housing-related factors were the main reason for 37.5% of the participants wanting to move [9]. Family- or disability-related factors were indicated as reasons for moving among younger individuals [9]. The findings also suggested that seeking better housing was indicated as the primary reason for moving among individuals aged 45 to 64 [9].

Another study by Botticello and colleagues suggested that approximately 25% of people moved within a five-year period, with long-distance moves being marginally less common than local ones [31]. While adults with an SCI/D moved less frequently compared to the general population, a significant number moved within five years [31]. More than half of these relocations were local, suggesting financial challenges, with about one-third of individuals moving to neighborhoods with high-poverty levels. [31]. A census area where less than 13% of the population resided below the poverty threshold was characterized as a low-poverty neighborhood [31]. Conversely, a census area where more than 13% of the population resided above the poverty threshold was characterized as a high-poverty neighborhood [31]. The findings across the two studies by Botticello and colleagues also suggested that the varying levels of SCI/D severity, age, and functional limitations may contribute to residential relocation [9,31].

## 4. Discussion

This scoping review examined the current state of evidence for housing across the continuum for individuals with an SCI/D and has provided several critical insights and identified notable gaps in the research pertaining to this topic. Overall, research on housing for the SCI/D population is relatively understudied, with only 36 studies identified in this review. When framed within the TOA, issues of accessibility presented the most substantial barriers for the SCI/D community, followed by acceptability and availability. Interestingly, one of the fewer explored domains across the studies was awareness (in 13 studies), which is an area that is worth further investigation, since there is evidence that higher levels of knowledge and awareness about the needs of individuals with disabilities can influence beliefs about the importance of barrier-free built environments [64]. Furthermore, individuals with an SCI/D experience challenges identifying and managing information in the community to support their health and social care needs [65]. Hence, more studies exploring ways to raise awareness to providers on the importance of accessible housing, including to those working in the housing sector, as well as providing information and resources to the SCI/D community, could lead to better accessible housing outcomes for this population.

In terms of design, the majority of studies were cross-sectional (*n* = 15), followed by those using qualitative methodologies (*n* = 9). Our review only identified one experimental study [47], one longitudinal prospective design [32], and one community-based RCT [30]. More longitudinal studies are needed to better understand how housing needs may change over time in the SCI/D community to obtain insights on the types of adaptations or support individuals require as they age. Furthermore, experimental designs might be beneficial for understanding how different interventions (e.g., home adaptations, educational resources, etc.) may lead to better housing outcomes. Importantly, there was a lack of studies employing community-based participatory research methods. People with disabilities have been traditionally excluded from meaningful engagement in housing research [19]. Future studies should aim to engage the SCI/D community to ensure that their housing needs and priorities are the ones being investigated.

The key findings across the studies indicated that inaccessible housing led to lower autonomy (especially in those with higher levels of injury), poorer QoL, and reduced community participation. Fifteen studies [10,34,36,38,39,41,43,44,48,49,50,52,55,57,59] explored the immediate outdoor space surrounding housing (e.g., ramps, entrances, sidewalks, backyard, etc.), which is also critical for assessing accessibility and safety, improving QoL, and enhancing autonomy among this population. Although studies exploring the relationships between housing and well-being used self-reported environmental measures, such as the CHIEF-SF and NEFI-SF, only three studies [48,49,52] used a validated objective measure to assess the degree of environmental barriers in the home, namely, the Housing Enabler. Further work using measures that can provide the degree of accessibility and that are linked to validated measures of health and well-being (e.g., 36-Item Short Form Survey, Life Satisfaction Questionnaire, etc.), would advance our understanding of how housing may influence outcomes in the SCI/D community.

Despite some community needs eventually being addressed, essential needs such as accessible housing, financial assistance, and support at home were often inadequately met in the first year post-discharge [13]. Dickson and colleagues’ report on the absence of post-discharge care after a return home [8] highlights the need for continued emotional and social support once individuals with an SCI/D return to their home. There is also a need for more research on housing type and design, particularly understanding the impacts of both indoor and outdoor environments, as well as the impact of housing type and design features on the potential for adaptability of a home for improved accessibility. There were eighteen studies describing the modifications people made to their homes, or the perceived impact of these modifications, such as installing ramps and modifying bathrooms, but many studies highlighted that people with an SCI/D still encounter significant barriers due to poorly designed environmental features and financial constraints. High costs [38] and lengthy installation processes remain significant obstacles [36], indicating a need for more affordable and efficient solutions. Moreover, while most participants reported improved autonomy from home modifications, those who had not adapted their homes continued to experience challenges with basic functional independence. Our review did not identify any studies that explored the economics of accessible housing for the SCI/D community. Future studies should make use of economic analyses given existing evidence [66,67], showcasing the cost savings to health and social care systems when investments in accessible housing are made.

Four studies [9,31,33,53] examined moving to some degree among the SCI/D population. One study revealed that residential mobility was lower in the SCI/D population than in the general population, but many individuals moved within a few years due to possible financial hardship or unstable housing [31]. Younger individuals and recently injured people had higher mobility rates compared to older, long-term injured participants [9]. As noted above, the use of more longitudinal studies may provide deeper insights on the factors that contribute to the need for moving following an SCI/D.

Many studies did not adequately address the racial and ethnic diversity of participants, nor did they thoroughly explore gender differences. Understanding these factors is crucial for comprehending how different groups experience and overcome housing challenges. A meta-analysis revealed that ethnic and gender discrimination is prevalent in the rental housing market, highlighting that minority and male applicants, particularly those with Arab/Muslim names, experience disadvantages [68]. The meta-analysis further indicated that discrimination by private landlords is more prevalent than that of real-estate agents [68]. It is recommended that future research explores gender and ethnic diversity in greater depth to determine how these identities influence housing.

In summary, this review emphasizes the need for further research to strengthen the evidence related to housing for individuals with an SCI/D. The majority of studies reviewed were conducted in Canada, the USA, and the UK, indicating a significant gap in research from other regions. Hence, more international research is needed to understand diverse contexts and improve housing solutions on a global scale. Additionally, examining the impact of social determinants of health, such as socioeconomic status, access to healthcare, and community resources, on the well-being of individuals with an SCI/D is essential. Addressing these research gaps will support the development of policies and interventions that enhance community integration, autonomy, and overall QoL for individuals with an SCI/D across diverse contexts.

## 5. Limitations

This scoping review was limited to articles published only in English, potentially excluding relevant studies in other languages. Research published in different languages may provide critical contextual information that is not included in English published sources. Additionally, some housing-related articles relevant to this patient population may not have been included, potentially leaving out essential information. Although searching both the academic and gray literature was a comprehensive approach, the gray literature may vary in rigor and is not subject to the peer review process, which could introduce potential biases to the SCI/D literature. Moreover, we revised the TOA and utilized it for a basic assessment; however, this approach was inherently subjective. While the subjectivity may limit the reliability of our findings, it did provide us with valuable insights into the housing situation of individuals with an SCI/D. Another limitation is the omission of the consultation exercise, as recommended by Levac et al. [22], who emphasize its critical nature for scoping reviews. The consultation exercise would enable knowledge transfer with interest holders, such as individuals with an SCI/D, their caregivers, and SCI/D organizations. Omitting the consultation exercise in this scoping review may have resulted in the exclusion of critical perspectives, potentially leading to incomplete or less relevant findings that do not fully explore the nuanced housing needs of the SCI/D population.

## 6. Conclusions

This review highlights a critical need for further research to better inform housing practices and policies for individuals with an SCI/D and suggests that there appears to be gaps in studies outside of Canada, the USA, and the UK. International research is essential to understand the diverse contexts and to develop effective housing solutions. Furthermore, exploring how social determinants of health impact the well-being of those with an SCI/D is crucial for generating evidence that can lead to meaningful changes in practice and policy in accessible housing. Addressing these gaps will support the creation of policies that enhance community integration, autonomy, and QoL for individuals with an SCI/D globally.

## Figures and Tables

**Table 1:** An adapted version of the TOA used to assess the included studies.

Dimensions	Definition	Examples	Criteria Used for TOA Assessment
Accessibility	Location	Accessible housing is within reasonable proximity for individuals, considering both time and distance, and meets the needs of people with an SCI/D.	Accessibility of the physical built environment of the home or neighborhood with respect to the interior and exterior of the home. Proximity of essential services, people, and places (e.g., grocery stores, community centers, medical clinics, etc.) near the homes of individuals with an SCI/D. Moving to a new home may be necessary if the current dwelling is physically inaccessible. Moving to an accessible home can significantly benefit individuals with an SCI/D by accommodating their mobility needs, enhancing their autonomy, and improving their overall QoL.
Availability	Availability of appropriate housing options for individuals with an SCI/D and the need for such housing for this population	Available housing has sufficient features and support services to meet the volume and specific needs of individuals with disabilities, such as an SCI/D, and their communities.	Perceptions of the home with respect to features and accommodations that meet the needs of individuals with an SCI/D. Availability of appropriate homes that meet individual needs. A lack of available homes that meet the specific needs of people with an SCI/D may result in the need for individuals to move to more suitable homes. Availability of resources (e.g., healthcare resources, education and informational media related to home accessibility, services, etc.) to assist with finding accessible homes or modifying homes. Availability of formal personal and home care needed to support accessibility in the home, and support with independent living and participation in activities of daily living (e.g., bathing, toileting, etc.) to help people manage an SCI/D.
Acceptability	Perspectives of individuals with an SCI/D	Acceptable housing responds to both the attitudes of the provider and the preferences and cultural considerations of individuals with an SCI/D.	Aligns with attitudes and preferences of people with an SCI/D (e.g., physically and socially inclusive, considers personal preferences, cultural and physical safety, and community connection); also fosters acceptance, well-being, and enhances autonomy, allowing individuals with an SCI/D to feel that their home is a suitable environment. A lack of acceptability in these areas may lead individuals to seek alternative housing options.
Affordability	Financial and incidental costs	Affordability examines the direct costs of accessible housing to the individual within the context of living with an SCI/D.	Direct costs related to housing (e.g., modifications), affordable housing, and housing management and maintenance. Mentions of financial difficulties related to housing (e.g., could be due to unemployment, low income, etc.) or financial support available to individuals with disabilities (e.g., pension, income assistance, etc.). Moving residences or dissatisfaction is related to financial constraints (e.g., current home is unaffordable and the cost of modifications may be too high). Moving may reduce financial strain.
Adequacy/Accommodation	Organization	An adequate resource/service is well organized to accept individuals with an SCI/D, and they are able to use the services.	Housing support services and care effectively meet the needs of the SCI/D community (e.g., housing satisfaction), ensuring that their physical and social environments support them. This includes effective housing management and maintenance that ensure individuals can fully utilize their living spaces and access necessary services without barriers. Enough suitable access to formal caregiving support. If applicable, did landlords accommodate the housing needs of individuals with an SCI/D?
Awareness	Communication and information	A service resource maintains awareness through effective communication and information strategies with relevant users (e.g., clinicians, individuals living with an SCI/D, and the broader community), including considerations of context and health literacy.	Awareness among individuals with an SCI/D and the broader community about both the availability of accessible housing options, including accessibility features, and the existence of related support services (e.g., housing- and health-related). Individuals with an SCI/D understand the types of services that are available to them, including how to access and utilize these services effectively. Residential moves identified as being related. Moving may be influenced by whether individuals have sufficient information about the housing options available to them and whether they are aware of all suitable choices within their area.

## Data Availability

All relevant data are presented in this review or as Supplementary Files.

## Supplementary Materials

The following supporting information can be downloaded at: https://www.mdpi.com/article/10.3390/healthcare12242537/s1, Table S1: Summary of 36 included studies. Reference [69] are cited in the supplementary materials.

## Appendix A

**Table A1:** Penchansky and Thomas’ TOA, with one additional update from Saurman [24,25].

Dimensions	Definition	Examples
Accessibility	Location	An accessible resource/service is within reasonable proximity of the consumer in terms of time and distance.
Availability	Supply and demand	An available resource/service has sufficient services and resources to meet the volume and needs of the consumers and communities served.
Acceptability	Consumer perception	An acceptable resource/service responds to the attitude of the provider and the consumer regarding the characteristics of the service and social or cultural concerns.
Affordability	Financial and incidental costs	Affordable resources examine the direct costs for both the service provider and the consumer.
Adequacy/Accommodation	Organization	An adequate resource/service is well organized to accept clients, and clients are able to use the services.
Awareness	Communication and information	A service/resource maintains awareness through effective communication and information strategies with relevant users (clinicians, patients, and the broader community), including considerations of context and health literacy.

## Appendix B. MEDLINE Search Strategy

# Query

1. exp spinal cord diseases/ or exp spinal cord injuries/ or Spinal Cord Neoplasms/
2. ((spine or spinal) adj3 (fracture* or wound* or trauma* or injur* or damag* or dysfunct* or compression* or tumor or tumour or lesion* or contusion* or laceration* or transection* or neoplasm*)).tw,kf.
3. SCI.tw,kf.
4. exp Hemiplegia/ or exp Quadriplegia/ or exp Paraplegia/
5. (paraplegi* or hemiplegi* or quadriplegi* or tetraplegi* or paresis or paraparesis or quadripares* or paraly?ed).tw,kf.
6. or/1–5
7. exp Housing Quality/ or exp Housing/ or exp Housing Instability/ or exp Housing for the Elderly/ or exp Public Housing/ or exp Architectural Accessibility/ or exp Independent Living/ or exp Residence Characteristics/
8. housing/ or almshouses/ or housing for the elderly/ or public housing/ or residential facilities/ or assisted living facilities/ or homes for the aged/
9. (almshouses or “assisted living facilities” or “homes for the aged” or “senior centers” or “intermediate care facilities” or “aging in place” or orphanages).tw,kf.
10. (house or houses or housing or condominium? or apartment* or townhouse* or townhome* or “built environment” or residential or architectur* or residential or dwelling or dwellings or lodging or lodgings or residence or residences or home or homes or rent or rents or duplex or “mobile home” or condo or bungalow or bungalows or “environmental barrier” or “environmental barriers” or “living arrangement” or “living situation” or “living arrangements” or “living situations”).tw,kf.
11. or/7–10 [**housing]
12. (“26681651” or “27636356” or “19528997” or “28359190”).ui.
13. 11 or 12
14. 6 and 13
15. exp animals/ not humans.sh.
16. 14 not 15

## Appendix C. Data Extraction Table

**General information**
Authors
Year of publication
Country of study origin
**Characteristics of included studies**
Aim(s) of study
Study design
Outcome measures
**Participants**
Sample size
Population studied
Level and severity of injury
Duration and/or range of injury
Mean age and/or age range of participants
Sex and/or gender of participants
Race and/or ethnicity of participants
Household income
Employment status
**Housing-related information**
Housing type
Living situation
Residence characteristics
Key findings and/or conclusions related to housing
Theory of Access construct(s)
