# Supplementary material for: A Scoping Review on the Influence of Housing on the Health and Well-Being of People with a Spinal Cord Injury/Dysfunction
Source: Healthcare (Basel). 2024 Dec 16;12(24):2537. doi: 10.3390/healthcare12242537 (PMC11675771; doi:10.3390/healthcare12242537)
Supplement: Supplementary file 1 [file healthcare-12-02537-s001.zip › healthcare-3257413-supplementary.pdf]

Table S1. Summary of 36 included studies.

Study, Location & Objective	Population	Housing Type, Living Situation, Residence Characteristics, & Outcome Measures	TOA Construct(s)	Key Findings
**Ahmad et al. [30]**<br>2013<br>Community-based randomized controlled trial (RCT)<br>Pakistan<br>To evaluate the impacts (successes) of home modification on QoL among individuals with SCI/D.	**Sample size** (N = 40): SCI/D (all participants used wheelchairs)<br><br>**Level & severity of injury:**<br>• Paraplegia (N = 40)<br><br>**Time since injury (in years):** Not reported<br><br>**Age (in years):**<br>• Experimental Group<br>• Mean: 33.66<br>• Range: 19 – 60<br>• Median: Not reported<br>• Control Group<br>• Mean: 31.57<br>• Range: 18 – 58<br>• Median: Not reported<br><br>**Sex/gender:**<br>• Male (n = 30)<br>• Female (n = 10)<br><br>**Race/ethnicity:** Not reported<br><br>**Household income:** Not reported<br><br>**Employment status:** Not reported	**Housing type:** Not reported<br><br>**Living situation:** Not reported<br><br>**Residence characteristics:** Not reported<br><br>**Outcome measures:**<br>• Modified Life Satisfaction (LiSAT) questionnaire (seven items [variables] were selected for this study)	Accessibility<br>Acceptability<br>Adequacy/Accommodation	• Home modifications improved QoL in the experimental group & reported a higher LiSAT score (33.32, p = 0.001), compared to both their pre-LiSAT score (19.11) & the LiSAT score of the control group (22.85).
**Botticello et al. [31]**<br>2022<br>Retrospective analysis<br>United States of America (USA)<br>To explore residential relocation in adults with SCI/D residing in the community & to identify factors concerning the	**Sample size** (N = 4,599): Traumatic SCI/D<br><br>**Level & severity of injury:**<br>• Complete tetraplegia (n = 800, 17.4%)<br>• Incomplete tetraplegia (n = 1,547, 33.6%)<br>• Complete paraplegia (n = 1,344, 29.2%)<br>• Incomplete paraplegia (n = 908, 19.8%)<br><br>**Time since injury (in years):**<br>• 1 – 5 (n = 1,252, 27.2%)<br>• 10 – 20 (n = 1,978, 43.0%)	**Housing type:** Not reported<br><br>**Living situation:** Not reported<br><br>**Residence characteristics:**<br>• Urban (n = 3,241, 70.5%)<br>• Suburban/rural (n = 1,358, 29.5%)	Acceptability<br>Affordability<br>Availability	• While residential mobility among adults with SCI/D was lower than in the general population, many relocated within five years, with 55.5% (n = 652) of people making local moves, gesturing to possible financial strain & unstable housing.<br><br>• A low-poverty neighbourhood was defined as a census tract with less than 13% of residents living in poverty, whereas a high-poverty area was identified as one with 13% or more of its population below the poverty threshold. About 25% of people moved within a 5-year period, with local moves (14.2%) being somewhat more frequent than long-distance ones (11.4%); 53.3% remained in low-poverty neighborhoods,

individual health & neighbourhood influencing individuals' likelihood of moving.	• 25 – 45 (n = 1,369, 29.8%)<br><br>**Age (in years):**<br>• Mean: 50.7 (SD = 14.0)<br>• Range: 23 – 94<br>• Median: Not reported<br><br>**Sex/gender:**<br>• Male (n = 3,693, 80.3%)<br>• Female (n = 906, 19.7%)<br><br>**Race/ethnicity:**<br>• Non-Hispanic White (n = 3,212, 70.2%)<br>• Non-Hispanic Black (n = 914, 20.0%)<br>• Hispanic (n = 356, 7.8%)<br>• Other (n = 91, 2.0%)<br><br>**Household income (annual, USD):**<br>• <$25,000 (n = 1,682, 36.6%)<br>• $25,000 – $49,999 (n = 1,394, 30.3%)<br>• $50,000 – $74,999 (n = 550, 12.0%)<br>• ≥ 75,000 (n = 973, 21.1%)<br><br>**Employment status:**<br>• Employed at time of first interview (time 1) (n = 1,178, 25.6%)<br>• Not working (time 1) (n = 3,421, 74.4%)	**Outcome measures:**<br>• National SCI Model Systems (SCIMS) Database<br>• American Community Survey (ACS) [neighbourhood characteristics were of interest]<br>• 'Moving' was regarded as a binary measure that indicated a change in residential location over a five-year period; 'move quality' was measured using poverty indicators in the census tract.		while 16.6% moved to a low-poverty neighbourhood & 8.9% moved to a high-poverty neighbourhood.<br><br>• 33% (n = 410) of the studied population moved to high-poverty neighborhoods & 21% (n = 974) of non-movers stayed in high poverty neighbourhoods proposing that many people are in potentially unsafe environments leading to community isolation & the deterioration of health status.<br><br>• Most participants lived in urban areas (70.5%), with the highest sample proportion in the Southern region of the United States (40.2%).<br><br>• Generally, neighbourhoods consisted of populations with moderate racial & ethnic diversity, high population density & a large percentage of owner-occupied housing.<br><br>• Across all logistic regression models, analysis revealed that individuals with fewer years post-SCI/D & lower-level injuries were more likely to relocate. Long-distance moves were higher among individuals with lower-level injuries, while individuals with incomplete tetraplegia were more likely to experience local moves. The length of time since injury has a negative relationship with move distance. The older individuals become, the less likely they were to relocate or move.<br><br>• Local moves & moving to a high-poverty neighbourhood were experienced by people from racial & ethnic minorities in comparison to those who self-identified as non-Hispanic & White.<br><br>• Factors such as education levels & income reduced the likelihood of moving to high-poverty neighbourhoods.<br><br>• Moving after SCI/D was less impacted by health status & more affected by individual & neighbourhood characteristics.
**Botticello et al. [9]**<br>2023<br>Cross-sectional observational study<br>USA<br>The main objectives of this study were to	**Sample size** (N = 690): SCI/D<br><br>**Level & severity of injury:**<br>• Paraplegia (n = 382; 55.4%)<br>• Tetraplegia (n = 308; 44.6%)<br>• Complete (n = 311; 45.6%)<br>• Incomplete (n = 379; 54.4%)	**Housing type:**<br>• House or condominium (n = 506, 73.3%)<br>• Other dwelling (n = 184; 26.7%)	Acceptability<br>Accessibility<br>Affordability<br>Availability<br>Awareness	• Annual move rate among people with SCI/D: 16.4%.<br><br>• The most common reasons for moving in the past 12 months were to obtain a home with improved accessibility (45.1%), improved housing quality (38.9%), more accessible places (28.3%), & wanting an independent household (27.4%), with housing-related factors being the primary reason for 37.5% of

(1) assess the overall & yearly rates of residential mobility following SCI/D & (2) assess the reasons for recent relocation among individuals with SCI/D. The secondary objective was to compare the annual moving rates & characteristics of movers with SCI/D to those of a general population sample.	**Time since injury (in years):**<br>• Mean: 14.2 (SD = 0.5)<br>• Range: 0 – 16+<br>• 2 years or less (n = 113, 16.4%)<br>• 3 – 5 years (n = 79, 11.5%)<br>• 6 – 10 years (n = 120, 17.4%)<br>• 11 – 15 years (n = 115, 16.7%)<br>• 16 + years (n = 263, 38.0%)<br>• Median: 11<br><br>**Age (in years):**<br>• Mean: 47.9 (SD = 14.1)<br>• Range: 18 – 65 +<br>• Median: Not reported<br><br>**Sex/gender:**<br>• Male (n = 542, 78.5%)<br>• Female (n = 148, 21.5%)<br><br>**Race/ethnicity:**<br>• Non-Hispanic White (n = 415, 60.8%)<br>• Non-Hispanic Black (n = 141, 20.6%)<br>• Hispanic (n = 94, 13.8%)<br>• Other (n = 264, 38.4%)<br><br>**Household income (annual, USD):**<br>• <$25,000, (n = 211, 34.0%)<br>• 25,000 – 49,999, (n = 141, 22.7%)<br>• 50,000 – 74,999, (n = 84, 13.6%)<br>• ≥ 75,000, (n = 184, 29.7%)<br>• Census tract household income (mean = 71,093, SD = 36,202.4)<br><br>**Employment status:**<br>• Employed for pay (n = 193, 28.1%)<br>• Not working (n = 497, 71.9%)	**Living situation**:<br>• Lives alone (n = 166, 24.1%)<br>• Lives with spouse/partner &/or children < 18 years old (n = 283, 41.0%)<br>• Lives with others (n = 241, 34.9%)<br>**Residence characteristics:**<br>• Home ownership (n = 328, 47.5%)<br>• Renting (n = 362, 52.5%)<br>• Receiving housing subsidy (n = 88, 12.8%)<br>• Not receiving housing subsidy (n = 602, 87.2%)<br><br>**Outcome measures:**<br>• Abbreviated SCIMS interview<br>• Questionnaire was administered via telephone by trained interviewers.<br>• Annual Social & Economic Supplement (ASEC) & Current Population Survey (CPS) were used to assess reasons for moving.		participants. 64.5% of participants reported moving at least once post-SCI/D, with an average of moving twice since their injury. Recently-injured individuals were more likely to move.<br><br>• Age was a major contributor to moving among this population. Housing was the most common reason for moving among study participants. It was indicated as the primary reason for moving among people aged 45 to 64. 55.6% of individuals aged 65 or older indicated disability as a reason for their recent move. Younger individuals indicated family- & disability-related reasons as more common reasons for moving.<br><br>• Younger individuals with SCI/D moved within the past year. Also, people who had recently been injured reported a higher rate of residential mobility than those with chronic SCI/D. Movers also tended to be at a lower socioeconomic level than non-movers & this study suggested that this may be attributed to the notion that some people may have financial resources for home modifications.
**Caro & Cruz [10]**<br>2020<br>Descriptive study<br>Brazil<br>To describe factors associated with functional mobility with regard to	**Sample size** (N = 11)**:** SCI/D<br><br>**Level & severity of injury:**<br>• Incomplete: (n = 10, 90.90%)<br>• Complete: (n = 1, 9.09%)<br>• Tetraplegia (n = 2, 18%)<br>• Paraplegia: Not reported	**Housing type:**<br>• House (n = 9, 81.81%)<br>• Apartment (n = 1, 9.09%)<br>• Farm (n = 1, 9.09%)<br><br>**Living situation:**	Acceptability<br>Accessibility<br>Adequacy/Accommodation<br>Availability	• Accessibility barriers were reported in the following areas: within their home (n = 9, 81.81%), sidewalk adjacent to their home (n = 10, 90.90%), & within their residential block (n = 11, 100%).<br><br>• The majority of participants indicated they were independent in terms of home mobility (100%; n = 11) on the sidewalk adjacent to their residence (100%; n = 11), within their block

wheelchair use in individuals with SCI/D.	**Time since injury (in years):**<br>• Median: 12.84 years<br><br>**Age (in years):**<br>• Mean: 38.27 (SD = 11.80)<br>• Range: 23 – 61<br>• Median: Not reported<br><br>**Sex/gender:**<br>• Male (n = 8, 72.72%)<br>• Female (n = 3, 27.27%)<br><br>**Race/ethnicity:** Not reported<br><br>**Household income:** Not reported<br><br>**Employment status:** 'Professional situation' was reported, in which participants fell into one or more of the following categories:<br>• Disability retirement: (n = 7, 63.63%)<br>• Work in the informal market: (n = 2, 18.18%)<br>• Student: (n = 19.09%)<br>• Continued Installment Benefit: (n = 1, 9.09%)<br>• Common retirement: (n = 1, 9.09%)	• Most participants resided with family members.<br><br>**Residence characteristics:**<br>• Urban (n = 10, 90.90%)<br>• Rural (n = 1, 9.09%)<br><br>**Outcome measures:**<br>• A Person Identification with Spinal Cord Injury Form, developed by the researcher, contained four main categories, including 'socio-demographic aspects, aspects related to the injury,' 'physical barriers for mobility in the home & surroundings & adaptations made' by individuals & 'mobility with the wheelchair used.'		(90.90%; n = 10), in accessible public or private spaces (90.90%; n = 10), in general public areas (90.90%; n = 10), on urban public transport (buses) (81.81%; n = 9) & for wheelchair transfers to surfaces at the same height (63.63%; n = 7).<br><br>• About 90.90% (n = 10) of participants made home & sidewalk adaptations, primarily installing lowered ramps or guides (n = 8), followed by bathroom modifications (n = 3), extension of doors/jamb (n = 3), non-slip on ramp (n = 1) & sill removal (n = 1).<br><br>• Common barriers reported in the home environment included sills (n = 10), uneven floors (n = 6), & holes (n = 4) at home; uneven floors (n = 11), steep ramps (n = 4) & inclined floors (n = 4) on the sidewalk; & similar issues in the surrounding block.
**Chan & Chan [32]**<br>2013<br>Longitudinal prospective design<br>China<br>To examine functional outcomes over a one-year period among Chinese individuals with SCI/D.	**Sample size** (N = 30)**:** SCI/D<br><br>**Level & severity of injury:**<br>• Traumatic (N = 30)<br>• Tetraplegia ASIA A/B/C (n = 10)<br>• Paraplegia ASIA A/B/C (n = 7)<br>• Tetraplegia ASIA D (n = 7)<br>• Paraplegia ASIA D (n = 6)<br><br>**Time since injury (in years):** Not reported<br><br>**Age (in years):**<br>• Mean:<br>• Tetraplegia ASIA A/B/C: 56.40 years (SD = 18.28 years)<br>• Paraplegia ASIA A/B/C: 35.43 years (SD = 12.47 years)<br>• Tetraplegia ASIA D: 51.29 years (SD = 14.94 years)	**Housing type:**<br>• Premorbid accommodation/home ownership<br>• 16 (53.33%) living in public housing estate<br>• 12 (40.00%) living in privately owned flat<br>• 3 (10.00%) living in rented flat<br>• Discharge accommodation<br>• 10 (33.33%) returning to previous public housing estate	Acceptability<br>Accessibility<br>Affordability<br>Availability	• All participants lived in the community prior to their SCI/D.<br><br>• Five participants were discharged to a private old-age home due to inadequate social support. For some participants (n = 11) occupational therapists conducted home assessments. Several participants (n = 3) were allocated new flats in public housing estates under the 'Hong Kong Housing Authority's compassionate re-housing policy'. These participants also received home modifications.<br><br>• Participants were prescribed various types of assistive equipment depending on their functional status, community environment & social support. Prescribed equipment included: wheelchairs, seating cushions, lifters, bathing/toileting aids, hospital beds & pressure relief mattress overlays.<br><br>• In the tetraplegia group (n = 10), 7 participants were prescribed manual wheelchairs & 2 were provided with

	• Paraplegia ASIA D: 35.84 years (SD = 19.92 years)<br>• Range: Not reported<br>• Median: Not reported<br><br>**Sex/gender:**<br>• Male (n = 23)<br>• Female (n = 7)<br>• Tetraplegia ASIA A/B/C: 7 males (23.33%); 3 females<br>• Paraplegia ASIA A/B/C: 5 males (16.67%); 2 females<br>• Tetraplegia ASIA D: 7 males (23.33%); no females<br>• Paraplegia ASIA D: 4 males (13.33%); 2 females<br><br>**Race/ethnicity:** Not reported<br><br>**Household income:** Not reported<br><br>**Employment status (after SCI/D):**<br>• Working (n = 1)<br>• Retired for age (n = 6)<br>• Retired for disability (n = 14)<br>• Homemaker (n = 1)<br>• Student (n = 1)<br>• Unemployed (n = 7)	• 11 (36.67%) returning to previous private/rented flat<br>• 3 (10.00%) moved (or re-housed) to public housing estate<br>• 5 (16.67%) transferred to old-aged homes<br><br>**Living situation:** Not reported<br><br>**Residence characteristics:** Not reported<br><br>**Outcome measures:**<br>• Functional Independence Measure (FIM)		power-driven wheelchairs. Most participants (n = 8) received seat cushions with air or foam-gel. Five participants received a commode/shower chair & a mobile hoist for transfers. Additionally, four hospital beds & seven pressure-relief mattress overlays were provided to this group.<br><br>• All participants in the paraplegia group (n = 7) received manual wheelchairs, seat cushions & a commode/shower chair, with the type provided (dependent or self-propelling) based on their social support & home environment.<br><br>• In the ASIA D group (n = 13), the types of assistive equipment varied among participants, with 6 participants receiving manual wheelchairs, 5 getting pressure-relief seat cushions & 3 receiving commode/shower chairs.
**Cleland et al. [7]**<br>2023<br>Qualitative - phenomenological approach<br>Australia<br>To explore the home automation experiences of individuals living with long term disability.	**Sample size** (N = 8)**:** Home automation users (n = 6) & their family members (n = 2)<br>• Tetraplegia (n = 2)<br>• Other physical disability (n = 4) & family members (n = 2)<br><br>**Level & severity of injury:**<br>• Tetraplegia (n = 3)<br>• Home automation users (n = 2)<br>• Family member of individual with tetraplegia (n = 1)<br><br>**Time since injury (in years):** Not reported<br><br>**Age (in years):**<br>• Mean: Not reported	**Housing type:** Not reported<br><br>**Living situation:**<br>• Home automation users (n = 6)<br>• Living with someone (n = 4)<br>• Living alone (n = 2)<br>• Family members (n = 2)<br>• Living with home automation user (n = 1)<br>• Living separately from	Acceptability<br>Accessibility<br>Adequacy/Accommodation<br>Availability<br>Awareness	• Years of experience using home automation: 1 – 32.<br><br>• Examples of home automation used by participants included, but were not limited to, lights, switches, door intercom, automatic door, heating & cooling, blinds, curtain & a fan.<br><br>• Two major categories identified:<br>• Benefits explored the experiences of participants using home automation & its impact. All participants highlighted that home automation allowed them to be independent within their homes. Participants' level of independence & dignity improved & they were less reliant on caregiving supports (including both formal/paid carers & family members).<br>• Home automation had a positive effect on several participants' mental health & wellbeing. It further promoted safety within their home, both physically & psychologically. Home automation enhanced

	• Range of all participants (including family members): 38 – 72<br>• Participants with tetraplegia were 50 & 60 years old.<br>• Family member of participant with tetraplegia was 58 years old.<br>• Median: Not reported<br><br>**Sex/gender:**<br>• Male (n = 4)<br>• Female (n = 4)<br><br>**Race/ethnicity:** Not reported<br><br>**Household income:** Not reported<br><br>**Employment status:** Not reported	home automation user (n = 1)<br><br>**Residence characteristics:** Not reported<br><br>**Outcome measures:**<br>• Semi-structured interviews		social & community interactions among participants, indicating they felt more energized & capable of safely leaving their homes.<br><br>• Challenges with using home automation included self-advocating to receive home automation, long waiting periods in the occupational therapist home assessment & installation process, frustrations when home automation did not work & the challenges experienced from being without home automation (e.g., when away from home on vacation).<br><br>• A participant with tetraplegia shared that installing home automation reduced their worries & improved their self-esteem.<br><br>• While participants reported that they felt more connected to the community, some revealed that home automation increased their independence at home but made them feel less independent in the community, which could deter them from participating in social activities, highlighting the need for more accessible & inclusive environments & the challenges of long installation processes.
**Coulombe et al. [33]**<br>2016<br>Mixed-methods<br>Canada<br>To describe the housing experiences of people with physical disabilities residing in family households.	**Sample size** (N = 31)**:** SCI/D<br><br>**Level & severity of injury:**<br>• Traumatic SCI/D (N = 31)<br>• Paraplegia (48.40%)<br>• Tetraplegia (51.60%)<br><br>**Time since injury (in years):**<br>• Mean: 8.02 (SD = 1.91)<br><br>**Age (in years):**<br>• Mean: 44.46 (SD = 14.93)<br>• Range: Not reported<br>• Median: Not reported<br><br>**Sex/gender:**<br>• Male (70.97%)<br>• Female (29.03%)<br><br>**Race/ethnicity:** Not reported<br><br>**Household income (CAD):**<br>• ≥$75,000 (53.84%)	**Housing type:**<br>• Single-family dwelling houses (73.33%)<br>• Semi-detached or terraced houses (16.67%)<br>• Apartments (10.00%)<br><br>**Living situation:** All participants resided with a family member.<br><br>**Residence characteristics:**<br>• Region<br>• Metropolitan areas (67.74%)<br>• Home ownership:<br>• Families owned their homes (93.55%)	Acceptability<br>Accessibility<br>Adequacy/Accommodation<br>Affordability	• The study showed a positive correlation between 'satisfying' home modifications & household income of the family.<br><br>• The more participants thought or believed that their chances of moving were high, the lower the index of neighbourhood assets were for them.<br><br>• There is an association between having received support to have home modifications & the positive potential of the home.<br><br>• Among the positive residential well-being (P-RWB) indices, only positive potential of the home significantly correlated with neighborhood assets, while unsatisfying home modifications & negative home potential were associated with desired neighborhood improvements in the negative residential wellbeing (N-RWB) indices.<br><br>• Overall, residents were more satisfied with their home modifications, as indicated by a higher index of 'satisfying' modifications compared to 'unsatisfying' ones.

	**Employment status:** Not reported; only the following was provided:<br>• Retired or taking care of family & home (61.29%)	• Moving: 48.48% of participants had moved since their injury<br>• Home modifications: A "complete modification" of the home was undertaken by 51.60% of participants<br><br>**Outcome measures:**<br>• Interviews<br>• Family Assessment Device (FAD) was used to measure family functioning & was completed by an adult in each participant household.		• When exploring the probability of moving in the next two years, some participants mentioned that they were unlikely to move, as their family was happy. However, some participants indicated that they were highly likely to move, due to difficulties in which the way the family functions, or perceived negative aspects within the home (e.g., size) and/or neighbourhood.
**Dickson et al. [8]**<br>2011<br>Qualitative research - interpretative phenomenological analysis<br>United Kingdom (UK)<br>To understand the lived experience of individuals with SCI/D.	**Sample size** (N = 17)**:** SCI/D<br><br>**Level & severity of injury:**<br>• C5, C6, or C7 SCI/D<br><br>**Time since injury (in years):**<br>• 17 months to 32 years<br><br>**Age (in years):**<br>• Mean: 46<br>• Range: 26 – 32<br>• Median: Not reported<br><br>**Sex/gender:**<br>• Male (n = 14)<br>• Female (n = 3)<br><br>**Race/ethnicity:** Not reported<br><br>**Household income:** Not reported<br><br>**Employment status:**<br>• Employed (n = 5)	**Housing type:** Not reported<br><br>**Living situation:** Not reported<br><br>**Residence characteristics:** Not reported<br><br>**Outcome measures:**<br>• Semi-structured interviews	Acceptability<br>Adequacy/Accommodation<br>Availability<br>Awareness	• Major themes, as reported by the authors, included "loss of camaraderie," "lack of post-discharge care," & "other people's reactions to SCI/D."<br><br>• Ongoing challenges in adapting to life at home following their stay at a rehabilitation centre was reported by many participants (n = 12). In particular, they struggled with the loss of companionship, which fostered a sense of connection among participants, in comparison to the isolation felt at home.<br><br>• The extended stay at the SCI/D unit created a fear of returning home, where participants would not have the support from other patients & access to healthcare providers. Everyone had an SCI/D at the rehabilitation centre, however, upon returning home, participants perceived themselves as distinct from both their pre-SCI/D identities & others, having to adapt to their new reality.<br><br>• Although eight participants regarded the SCI/D unit as their "home," three expressed feelings of being institutionalized, highlighting their frustration with adjusting back to their own homes. They described a sense of not truly "belonging" there as they had in the rehabilitation centre, making their transition to home life more challenging. Some participants did not

				experience the same level of safety & security as they had within the unit.<br><br>• Although participants (n = 10) had positive care experiences in the SCI/D unit, they expressed feelings of being abandoned after discharge, citing a lack of ongoing support for both physical & psychological needs. Many reported facing substantial gaps in psychological care during their rehabilitation & when transitioning back home, leading to unmet needs. Psychological support should be routinely available, regardless of whether individuals actively seek or request it, according to participants.<br><br>• Ten participants described negative encounters related to their use of a wheelchair, highlighting a strong sense of being perceived as different. This perception resulted in feelings of inferiority compared to individuals without disabilities, which in turn intensified their experiences of frustration, isolation & loneliness.<br><br>• This study emphasized the importance of post-SCI/D care to support individuals' psychosocial well-being & transition outside of the SCI/D unit.
**Dorjbal et al. [34]**<br>2020<br>Qualitative<br>Mongolia<br>To determine environmental barriers & their perceived impacts by individuals with SCI/D.	**Sample size** (N = 16)**:** SCI/D<br><br>**Level & severity of injury:**<br>• Paraplegia (n = 11)<br>• Tetraplegia (n = 5)<br>• Incomplete (n = 8)<br>• Complete (n = 8)<br>• Traumatic (N = 16)<br><br>**Time since injury (in years):** Not reported<br>• Date of injury: April 2001 – September 2011<br><br>**Age (in years):**<br>• Mean: Not reported<br>• Range: 25 – 45<br>• Median: Not reported<br><br>**Sex/gender:**<br>• Male (n = 9)	**Housing type:** Not reported<br><br>**Living situation:** Not reported<br><br>**Residence characteristics:**<br>• Living area<br>◦ Urban (n = 8)<br>◦ Rural (n = 8)<br><br>**Outcome measures:**<br>• Questionnaire<br>• Semi-structured interviews – elements of the International Classification of Functioning, Disability & Health were used as a	Acceptability<br>Accessibility<br>Adequacy/Accommodation<br>Availability	• Participants reported seven major barriers for mobility & other daily activities, including: (1) insufficient access to the physical environment; (2) lack of wheelchair-accessible transportation; (3) unfavourable societal attitudes; (4) insufficient healthcare & rehabilitation services; (5) limited access to assistive devices & medications; (6) constrained financial resources for healthcare; & (7) misclassification of disabilities in legislation.<br><br>• All participants reported barriers in their physical environment including entrances & exits of buildings & restrooms.<br><br>• Barriers to entrances included lack of ramps & lifts, while barriers to restrooms included a lack of wheelchair-accessible toilets & bathrooms. Participants expressed that they have to receive support from others (e.g., family members, neighbours & strangers) due to these accessibility barriers.

	• Female (n = 7)<br><br>**Race/ethnicity:** Not reported<br><br>**Household income:** Not reported<br><br>**Employment status:**<br>• Unemployed (n = 10)<br>• Volunteer work (n = 2)<br>• Self-employed (n = 4)	framework to structure the interview guide		
**Farahani et al. [12]**<br>2021<br>Qualitative research (conventional content analysis)<br>Iran<br>To explore the experiences of people with SCI/D concerning the factors that support their efforts to regain independence during the transition to the community from the hospital.	**Sample size** (N = 15)**:** SCI/D<br><br>**Level & severity of injury:**<br>• Complete (n = 6)<br>• Incomplete (n = 9)<br><br>**Time since injury (in years):** Not reported<br><br>**Age (in years):**<br>• Mean: 33.8<br>• Range: 25 – 44<br>• Median: Not reported<br><br>**Sex/gender:**<br>• Male (n = 9)<br>• Female (n = 6)<br><br>**Race/ethnicity:** Not reported<br><br>**Household income:** Not reported<br><br>**Employment status:** Not reported	**Housing type**: Not reported<br><br>**Living situation:** Not reported<br><br>**Residence characteristics:** Not reported<br><br>**Outcome measures:**<br>• Semi-structured interviews	Acceptability<br>Accessibility<br>Adequacy/Accommodation<br>Availability<br>Awareness	• Four main elements were identified in helping people with SCI/D regain their independence, including "self-management, social support, access to facilities & resources & spiritual & religious beliefs."<br><br>• Many individuals sought information to increase their SCI/D-related knowledge (e.g., Internet, books, physician), as they stated that it helped them to better manage their SCI/D.<br><br>• Participants also received support from others (e.g., family & friends, healthcare professionals, & peers).<br><br>• Home modifications (e.g., bathroom modifications) substantially enhanced individuals' autonomy. As well, sufficient access to rehabilitation facilities, as well as mobility equipment, was reported as a "great help" for their independence.<br><br>• Some participants noted that moving to more appropriate homes was necessary due to accessibility barriers in their current homes (e.g., absence of a lift in an apartment).<br><br>• Mobility equipment & assistive devices (e.g., wheelchairs, walkers, etc.), were used by many individuals, improving their autonomy.<br><br>• Religious beliefs were revealed to aid individuals in accepting & managing their SCI/D, as they sought to regain independence.
**Gulati et al. [35]**<br>2011<br>Retrospective cohort study<br>UK<br>To describe the functional outcomes	**Sample size** (N = 39): Traumatic SCI/D<br>• Deceased (n = 9) after 44 days of hospital admission (median value)<br>• Surviving (n = 30)<br><br>**Level & severity of injury (surviving, n = 30):**<br>• Complete (n = 6, 20%)	**Housing type:**<br>• Discharge locations (n = 30):<br>• Returned home (n = 11, 37%)<br>• Nursing home/communit	Availability	• 11 individuals (37%) with incomplete SCI/D were able to return home. They achieved greater FIM scores at both the beginning of rehabilitation & at discharge when compared to those who were transferred to a nursing home or another hospital ($P < 0.01$ & $P < 0.0001$, respectively).

& discharge locations of elderly individuals who have incurred a traumatic SCI/D.	• Incomplete (n = 24, 80%)<br>• Traumatic (n = 30)<br>• Injury location<br>• Cervical (n = 21, 70%)<br>• Thoracic (n = 3, 10%)<br>• Lumbar (n = 6, 20%)<br>• ASIA Classification (at admission)<br>• AIS A (n = 6)<br>• AIS B (n = 2)<br>• AIS C (n = 9)<br>• AIS D (n = 13)<br>• AIS E (n = 0)<br>• ASIA Classification (at discharge)<br>• AIS A (n = 4)<br>• AIS B (n = 0)<br>• AIS C (n = 4)<br>• AIS D (n = 21)<br>• AIS E (n = 1)<br><br>**Time since injury (in years):** Not reported<br><br>**Age (in years) (surviving, n = 30):**<br>• Mean: 73<br>• Range: 65-88<br>• Median: Not reported<br><br>**Sex/gender:** Not reported<br><br>**Race/ethnicity:** Not reported<br><br>**Household income:** Not reported<br><br>**Employment status:** Not reported	y hospitals (n = 10, 33%)<br>• Referring hospital (n = 9, 30%)<br><br>**Living situation:** Not reported<br><br>**Residence characteristics:** Not reported<br><br>**Outcome measures:**<br>• American Spinal Cord Injury Association (ASIA) impairment scale (AIS) & ASIA motor scores at the point of injury & prior to discharge were documented<br>• Functional Independence Measure Score (FIM)		• Individuals with complete SCI/D (AIS A) did not return to their initial homes, suggesting that age & injury severity influence discharge outcomes.<br><br>• Improvements in AIS classifications were observed among 12 individuals at discharge compared to admission, which the authors indicate that some level of motor improvement could be observed in elderly individuals with considerable paralysis.<br>• AIS A to AIS C (n = 2, 33%)<br>• AIS B to AIS D (n = 2, 100%)<br>• AIS C to AIS D (n = 7, 77%)<br>• AIS D to AIS E (n = 1, 8%)
**Hall et al. [36]**<br>2021<br>Qualitative research - grounded theory<br>USA<br>To explore the lived experiences of people post-SCI/D & the perspectives of SCI/D therapists**.**	**Sample size** (N = 10)**:** Traumatic SCI/D<br><br>**Level & severity of injury:**<br>• Traumatic (N = 10)<br><br>**Time since injury (in years):**<br>• Mean: Not reported<br>• Range: 1 - 20 or more years<br>• Median: Not reported<br><br>**Age (in years):**<br>• Mean: Not reported<br>• Range: 29 - 65 years	**Housing type:** Not reported<br><br>**Living situation:** Not reported<br><br>**Residence characteristics:** Not reported<br><br>**Outcome measures:**<br>• Semi-structured interviews	Acceptability<br>Accessibility<br>Affordability<br>Availability<br>Awareness	• Most participants felt the necessity to modify their homes & vehicles, despite the associated costs. The most common modifications included adding ramps, widening doorways & renovating bathrooms.<br><br>• Participants identified challenges in their communities, noting that inaccessible sidewalks & irregular road surfaces obstructed their ability to access places, such as school & work.<br><br>• Individuals without family financial support indicated that this restricted their capacity to seek various services, home modifications, assistive devices & transportation expenses.

	• Median: Not reported<br><br>**Sex/gender:**<br>• Male (n = 9)<br>• Female (n = 1)<br><br>**Race/ethnicity:** Not reported<br><br>**Household income:** Not reported<br><br>**Employment status:**<br>• Employed (n = 3)			They also noted that the process of applying for financial assistance was challenging due to its lengthy application processes (paperwork & waiting time).<br><br>• Participants expressed uncertainty about what to expect after leaving the hospital & indicated a need for more information on various activities, including, but not limited to, job seeking, mobility & accessibility accommodations, financial resources & daily activities (e.g., meal preparation, using the restroom, training in wheelchair use, etc.). They also noted that they did not receive sufficient information about maintaining their "overall health" (e.g., managing bladder & bowel functions, urinary catheter care, etc.), as the focus was primarily on their injury.<br><br>• Personal motivation & strong relationships with healthcare providers were also considered vital. A concern expressed was the challenge of finding local healthcare providers who have the expertise & understanding necessary to support people with SCI/D.
**Heinemann et al. [37]**<br>2016<br>Adopted mixed-method qualitative & quantitative approach<br>USA<br>To create a metric for natural environment & human-made alterations, & to assess how perceived barriers impact health-related QoL.	**Sample size** (N = 604, n = 570 completed built & natural environment (BNE) items)**:**<br>• SCI/D (n = 193)<br>• CVA (stroke) (n = 203)<br>• Traumatic Brain Injury (TBI) (n = 174)<br><br>**Level & severity of injury:** Not reported<br><br>**Time since injury (in years) (SCI/D):**<br>• Mean: 12.2 (SD = 10.1)<br><br>**Age (in years) (n = 193):**<br>• Mean: 45.3 (SD = 13.9)<br>• Range: Not reported<br>• Median: Not reported<br><br>**Sex/gender (n = 193):**<br>• Male (n = 155, 80%)<br>• Female (n = 38, 20%)<br><br>**Race/ethnicity (N = 192):**<br>• Hispanic any race (n = 18, 9%)<br>• Non-Hispanic white (n = 117, 61%)<br>• Non-Hispanic black (n = 52, 27%)<br>• Non-Hispanic other (n = 5, 3%)	**Housing type:** Not reported<br><br>**Living situation (N = 200):**<br>• Living alone (SCI/D) (n = 44, 22%)<br>• Living with others (SCI/D) (n = 156, 78%)<br><br>**Residence characteristics:** Not reported<br><br>**Outcome measures:**<br>• Community participation indicators (CPIs)<br>• Patient-reported outcomes measurement information system (PROMIS) methodology<br>• Framework used: World Health Organization (WHO)	Acceptability<br>Accessibility	• 88% (n = 169) of participants with SCI/D were wheelchair users, whereas 12% (n = 24) were not.<br><br>• The evaluation showed that out of 18 comparisons between wheelchair users and nonusers across the study (e.g., SCI/D, CVA, TBI), four were statistically significant ($p < 0.003$ with Bonferroni correction, Mantel Chi-square test). Wheelchair users were more likely to report difficulties with "moving around outdoors," "getting in stores," "moving around your home" & "feeling (un)safe in your home during an emergency" compared to nonusers.<br><br>• Environmental barriers significantly impact individuals with moderate-to-severe physical or cognitive disabilities. Physically accessible environments are advantageous for those using wheelchairs & assistive devices.

	**Household income:** Not reported<br><br>**Employment status (N = 191):**<br>• Employed (n = 52, 27%)<br>• Not employed (n = 139, 73%)	International Classification of Functioning, Disability & Health (ICF) framework		
**Hertig-Godeschalk et al. [38]**<br>2018<br>Cross-sectional study<br>Switzerland<br><br>The authors stated three aims: (1) to assess the availability of home adaptations based on self-reports, (2) to analyze the usage patterns of various home adaptations by calculating a measure of their overall availability & (3) to assess which home adaptations individuals feel they need but do not have, indicating they perceive this as an unmet need.	**Sample size** (N = 482): Chronic SCI/D<br><br>**Level & severity of injury:**<br>• Traumatic (n = 366, 76.7%)<br>• Non-traumatic (n = 111, 23.3%)<br>• Incomplete paraplegia (n = 190, 39.7%)<br>• Complete paraplegia (n = 143, 29.9%)<br>• Incomplete tetraplegia (n = 103, 21.5%)<br>• Complete tetraplegia (n = 42, 8.8%)<br><br>**Time since injury (in years):**<br>• 0 – 5 (n = 8, 2.3%)<br>• 6 – 15 (n = 143, 40.6%)<br>• 16 – 25 (n = 88, 25.0%)<br>• 26 + (n = 113, 32.1%)<br>• Mean: 16.2 (SD = 13.2)<br>• Median: 12<br><br>**Age (in years):**<br>• Mean: 55.2 (SD = 15.0)<br>• Range:<br>o 16 – 30 (n = 28, 5.8%)<br>o 31 – 45 (n = 103, 21.4%)<br>o 46 – 60 (n = 148, 30.7%)<br>o 61 – 75 (n = 171 35.5%,)<br>o 76 + (n = 32, 6.6%)<br>• Median: 44<br><br>**Sex/gender:**<br>• Male (n = 345, 71.6%)<br>• Female (n = 137, 28.4%)<br><br>**Race/ethnicity:** Not reported<br><br>**Household income:** Not reported<br><br>**Employment status:** Not reported	**Housing type:** Not reported<br><br>**Living situation:**<br>• Living with someone else (n = 363, 76.7%)<br>• Living alone (n = 110, 23.3%)<br><br>**Residence characteristics:**<br>• Without formal support/care in a non-adapted home (n = 166, 37.8%)<br>• Without formal support/care in an adapted home (n = 129, 29.4%)<br>• With formal support/care in an adapted home (n = 136, 31.0%)<br>• With formal/support care in a community home (n = 8, 1.8%)<br>• Participants living in an adapted home (62%)<br><br>**Outcome measures:**<br>• Swiss Spinal Cord Injury (SwiSCI) Community Survey (for self-reported availability and unaddressed need for home adaptations)<br>• Spinal Cord Independence	Acceptability<br>Accessibility<br>Adequacy/Accommodation<br>Affordability<br>Availability	• Among the participants (n = 255) who answered questions about home adaptations, the median number of home adaptations reported was 3. 14.9% of participants expressed not having any home adaptations, 34.1% expressed having 1-2 home adaptations, 32.9% expressed having 3-4 home adaptations & 18.0% expressed having 5 or more.<br><br>• The most common home adaptation that participants had was a wheelchair accessible shower, a grab bar close to the toilet & a ramp; as reported by 62.7%, 56.7% & 45.0% of participants, respectively. Combinations most reported were a grab bar next to the toilet paired with a wheelchair-accessible shower (32.2%) & a wheelchair-accessible shower with a wheelchair-accessible kitchen worktop (25.5%) among individuals who answers questions related to home adaptations.<br><br>• Additionally, participants (n = 89) answered questions about the home adaptations (81 responses) available to them that were not covered previously in the study questionnaire. Available housing adaptations included a wheelchair-accessible parking lot or garage, noted in seven of the 81 responses (8.6%), as well as a wheelchair-accessible garden or balcony (8.6%).<br><br>• Participants were asked what home adaptations they were in need of that was not covered previously in the study questionnaire, 44 had answered & 6 (13.6%) of the responses shared the need for adaptations such as blinds, door & windows that are controlled electronically, 5 (11.4%) shared their need for access to their garden or balcony & 4 (9.1%) shared the need for kitchen appliances.<br>• The two home adaptations most commonly identified as unmet needs were adjustable kitchen worktops, reported by 78.7% of participants & adjustable kitchen cabinets, noted by 75.7%.<br><br>• There were no significant differences in the availability of home adaptations related to the sex, cause of injury, or

		Measure (SCIM-SR, items 12 & 13)		time since injury. Additionally, living arrangements did not significantly impact the availability of home adaptations; however, participants living with others tended to have fewer kitchen modifications than those living independently.<br><br>• Individuals who used wheelchairs had significantly more access to adaptations like stair lifts, accessible showers, & ramps.<br><br>• Significant differences with regard to SCI/D severity were observed; individuals with complete SCI/D had more home adaptations.<br><br>• Age appeared to influence some adaptations, with older individuals having more grab bars installed outside bathrooms & fewer accessible showers.<br><br>• While there were no significant differences in unmet needs related to financial hardship, participants experiencing some or severe financial difficulties reported a greater unmet need for more expensive home adaptations, including stair lifts, automatic door openers & wheelchair-accessible showers.
**Jeawon et al. [39]**<br>2023<br>Mixed-methods<br>Canada<br>(1) to investigate the relationships between subjective QoL & various socio-demographic factors among individuals with SCI/D & (2) to assess the experiences of individuals with differing QoL levels (low, moderate & high).	**Sample size** (N = 24)**:** Incomplete SCI/D<br><br>**Level & severity of injury:**<br>• Incomplete (N = 24)<br>• Traumatic (n = 14, 58.30%)<br>• Non-traumatic (n = 6, 25.00%)<br>• Did not know (n = 4, 16.7%)<br>• Cervical (n = 9, 37.50%)<br>• Thoracic (n = 6, 25.00%)<br>• Lumbar (n = 9, 37.50%)<br><br>**Time since injury (in years):**<br>• Mean: 21.88 (SD = 16.30)<br>• Range: 2 – 52<br><br>**Age (in years):**<br>• Mean: 54.60 (SD = 14.60)<br>• Range: 27 – 72<br>• Median: Not reported<br><br>**Sex/gender:**	**Housing type:** Not reported<br><br>**Living situation:**<br>• Live alone (n = 8, 33.33%)<br>• Live with somebody (n = 16, 66.67%)<br>**Residence characteristics:**<br>• Location of residence<br>• City (n = 13, 54.16%)<br>• Suburban (n = 7, 29.17%)<br>• Rural (n = 4, 16.67%)<br><br>**Outcome measures:**<br>• Demographic survey<br>• Functional Ambulation Measure	Acceptability<br>Accessibility<br>Adequacy/Accommodation<br>Affordability	• Among the participants categorized by QoL levels, the majority in the low quality of life group were female, while most in the high quality of life group were male.<br><br>• A relationship between living situation & sex was noted. Low QoL was noted in both males & females living alone. In contrast, males living with others reported the highest QoL, whereas females in the same situation reported the lowest QoL.<br><br>• Individuals in the low QoL group encountered more barriers compared to the high QoL group. The high QoL group experienced more facilitators & few barriers. The moderate QoL group exhibited a balance between QoL-related barriers & facilitators.<br><br>• Six perceived factors negatively influencing QoL in the low QoL group include, “feeling socially isolated,” financial worries, “feeling judged,” “feeling left out due to city infrastructure not being wheelchair or walker-friendly,” attributing “medical negligence” for their

	• Male (n = 13, 54.16%)<br>• Female (n = 11, 45.83%)<br><br>**Race/ethnicity:** Not reported<br><br>**Household income (gross):**<br>• Income range: less than $14,999 to greater than $75,000<br>• Ten participants preferred not to answer<br><br>**Employment status:**<br>• Employed (n = 4, 16.67%)<br>• Not employed (n = 20, 83.33%)	• Life Satisfaction Questionnaire—11<br>• Spinal Cord Independence Measure—III<br>• 12-Item Short-Form Survey<br>• Semi-structured interviews		SCI/D & worsening mobility. Four participants reported that their restricted income (unemployed, & reliant on disability pensions) resulted in unmet medical needs & inability to obtain essential assistive devices such as foot orthoses or powered wheelchairs. Financial concerns with regard to costly medical equipment were indicated among by low QoL participants. A factor positively influencing QoL was the "ability to adapt to injury" was indicated by one participant.<br><br>• The majority of participants in the low QoL group also experienced barriers in the built environment due to elements, including, but not limited to, inaccessible sidewalks, an absence of curb cuts, hilly areas & stairs.<br><br>• Adapting to life post-injury, valuing family & social support & maintaining financial stability were identified as three critical factors that positively influenced QoL in the moderate QoL group. Seven participants noted the use of adaptive devices & environmental adaptations, and the majority of participants in the moderate QoL group (n = 5) did not report significant financial barriers. "Feeling judged" & "decreasing mobility" were reported as factors that negatively affected participants in the moderate QoL group.<br><br>• Participants in the high QoL group identified several factors that enhance their QoL. These included showing resilience, adapting to life post-SCI/D, valuing their family & social networks, feeling included in SCI/D & community activities, experiencing minimal differences in daily life during COVID-19 restrictions & having financial security.<br><br>• Among participants with low QoL, 80% identified the environment or infrastructure as hindrances to mobility within the community.
**Kennedy et al. [40]**<br>2010<br>Multi-centre, single cohort study<br>UK<br>To determine critical areas where needs are unmet, to explore	**Sample size** (N = 80)**:** SCI/D<br><br>**Level & severity of injury:**<br>• Complete tetraplegia (n = 8, 10%)<br>• Incomplete tetraplegia (n = 23, 29%)<br>• Complete paraplegia (n = 17, 21%)<br>• Incomplete paraplegia (n = 23, 29%)<br>• Did not know (n = 9, 11%)	**Housing type:** Not reported<br><br>**Living situation:** Not reported<br><br>**Residence characteristics:** Not reported	Acceptability<br>Accessibility<br>Adequacy/Accommodation<br>Availability<br>Awareness	• The CHIEF-SF was used to evaluate the impact of environmental barriers. Among the various categories assessed, the Physical & Structural category had the highest mean score at 2.24 (SD = 2.27), compared to other categories such as Attitudes & Support, Services & Assistance & Work & School. Overall, these environmental barriers appeared to have a minimal effect on activities among participants in this study.

discrepancies in the delivery of rehabilitation services & perceived environmental obstacles affecting individuals' participation in their communities.	**Time since injury (in years):** Not reported<br><br>**Age (in years):**<br>• Mean: 50.37 years<br>• Range: 18 – 81<br>• Median: Not reported<br><br>**Sex/gender:** Not reported<br>• Ratio of men to women: 2:1<br><br>**Race/ethnicity:** Not reported<br><br>**Household income:** Not reported<br><br>**Employment status:**<br>• Employed at time of the study (n = 26, 32%)<br>• Actively looking for employment (7.6%)	**Outcome measures:**<br>• Demographic questions<br>• Functional Independence Measure (FIM)<br>• Hospital Anxiety & Depression Scale<br>• Craig Hospital Inventory of Environmental Factors (CHIEF-SF)<br>• Craig Handicap & Assessment Reporting Technique (CHART)<br>• Pain Numerical Rating Scale<br>• Life Satisfaction Questionnaire<br>• Current Needs Assessment<br>• Secondary Complication Screening Instrument<br>• Female Sexual Function Index<br>• Open questions (developed by the researcher)	• Challenges after discharge, primarily related to accommodation & adaptations (29%), including living in unsuitable family homes or spending time in a care facility before moving to a new residence were reported by a significant portion of participants (67.9%). Additionally, 25% of respondents faced difficulties with psychological & social adjustments, expressing feelings of abandonment & anxiety. Problems with rehabilitation services were noted by 22% of participants, particularly in terms of insufficient support during the transition home. Furthermore, delays in obtaining essential adaptations were reported by 49% of participants.<br><br>• Challenges in the transition were primarily due to insufficient resources, with adaptations (35%), equipment (27%) & limited access (19%) being the most significant factors.<br><br>• Participants identified independence as a crucial factor (16%) when discussing quality of life, highlighting aspects such as, “being able to live independently in my own home.”<br><br>• Accommodation & access (54%), equipment (33%) & community & rehabilitation (13%) were identified as the most frequently mentioned themes in response to an open-ended question asking, "What resources were you lacking that made the transition difficult?"<br><br>• Responses to open-ended questions also revealed that environmental challenges hindered the transition to living in the community.<br><br>• Beyond challenges related to accommodations, adaptations & the availability of equipment, this study found that secondary conditions & pain significantly influence independence & activity of individuals with SCI/D.<br><br>• The authors suggest that, prior to discharge, individuals should receive more information about equipment & supplies, as well as timelines for adaptations, which could enhance the psychological well-being of this population & support them.

**Khalili et al. [41]**<br>2023<br>Cross-sectional study<br>Canada<br>(1) To assess & compare the perceived autonomy among wheeled mobility assistive devices (WMAD) users in five distinct environments.<br>(2) To assess how factors related to personal, environmental, & assistive device aspects affect the perceived autonomy of WMAD users in these environments.	**Sample size** (N = 123)**:**<br>• SCI/D (n = 76, 61.8%)<br>• Congenital Spinal Cord Abnormality (e.g., Spina Bifida) (n = 10, 8.1%)<br>• Multiple Sclerosis (n = 6, 4.9%)<br>• Cerebral Palsy (n = 5, 4.1%)<br>• Arthritis (n = 4, 3.3%)<br>• Post-Polio (n = 4, 3.3%)<br>• Muscular Dystrophy (n = 2, 1.6%)<br>• Stroke (n = 1, 0.8%)<br>• Other (e.g., Amputee, Pelvic Trauma) (n = 15, 12.2%)<br><br>**Level & severity of injury:** Not reported<br><br>**Time since injury (in years):** Not reported<br><br>**Age (in years):**<br>• Mean: 49.1 (SD = 13.3)<br>• Range: 24 – 77<br>• Median: Not reported<br><br>**Sex/gender:**<br>• Male (n = 51)<br>• Female (n = 72)<br><br>**Race/ethnicity:** Not reported<br><br>**Household income:** Not reported<br><br>**Employment status:** Not reported	**Housing type:** Not reported<br><br>**Living situation:** Not reported<br><br>**Residence characteristics:** Not reported<br><br>**Outcome measures:**<br>• Semi-structured online survey with closed- & open-ended questions	Acceptability<br>Accessibility<br>Adequacy/Accommodation<br>Availability	• The five environments were ranked by satisfaction with regards to autonomy from highest to lowest as follows: Home Environment, Buildings Outside of the Home Environment & Transportation, Outdoor Built Environment, & Outdoor Natural Environment.<br><br>• Overall satisfaction with autonomy during activities in the Outdoor Natural Environment was significantly lower than the other four environments, according to a Wilcoxon signed-rank test for paired comparisons. No significant differences in overall satisfaction with regard to autonomy were noted among the other environments.<br><br>• The Cronbach's alpha values for perceived satisfaction with specific environmental factors ranged from 0.91 to 0.94 across the five environments. "Health conditions," "reach," & navigating "narrow spaces" were consistent across the five environments, but factors specific to each of the environments were also observed, such as maneuvering stairs & curbs (low satisfaction in Outdoor Built Environments, Home Environment, & Buildings Outside the Home).<br><br>• Participants frequently reported that home modifications significantly influence autonomy, with common issues in the home environment including doorways, level changes/stairs & reach difficulties. Challenges inside buildings external to the home environment included navigating doorways, navigating narrow spaces & soft surfaces (e.g., carpets). The outdoor built environment was cited as having more challenges than the home or other buildings, with concerns such as level changes/stairs, weather, & traversing natural environments (e.g., grass, sand, etc.).<br><br>• Users of WMADs typically reported the highest levels of satisfaction with their autonomy in home environments, linked to factors such as home modifications & specific features of WMADs, such as elevation capabilities. According to the regression analysis, the ability to navigate narrow spaces (e.g., doorways, small areas) was a significant predictor of overall fulfillment with autonomy in the home. Additionally, similar concerns about reach & maneuverability were noted in buildings outside the home.

				• While WMAD users reported low satisfaction with autonomy related to specific factors (e.g., navigating stairs, curbs, etc.), these factors did not play a significant role in their overall feeling of independence, & may be attributed to their expectations concerning their devices & capabilities. Conversely, factors that notably influenced autonomy included "maneuverability in tight spaces, maneuverability on various terrains, safety & the distance traveled." These factors (one or more) could allow for prediction of autonomy in each setting.
**Labbé et al. [43]**<br>2016<br>Q-methodology<br>Canada<br>To determine the housing priorities of individuals living with SCI/D & their family members.	**Sample size** (N = 59): SCI/D (n = 29) & household members (n = 30)<br><br>**Level & severity of injury:**<br>• Paraplegia (n = 14, 48.27%)<br>• Tetraplegia (n = 14, 48.27%)<br>• Not specified (n = 1, 3.46%)<br><br>• Traumatic (n = 24)<br>• Non-traumatic (n = 4)<br>• Not specified (n = 2)<br><br>**Time since injury (in years):**<br>• Mean: 7.93 (SD = 2.18)<br>• Range: 5.38 – 11.78<br><br>**Age (in years):**<br>• Mean (SCI/D): 44.96 (SD = 15.29)<br>• Range (SCI/D): 23.14 – 69.61<br>• Mean (household members): 51.43 (SD = 15.15)<br>• Range (household members): 23.67 – 80.89<br>• Median: Not reported<br><br>**Sex/gender:**<br>• SCI/D<br>• Male (n = 21, 72.41%)<br>• Female (n = 8, 27.59%)<br>• Household members<br>• Male (n = 8, 26.67%)<br>• Female (n = 22, 73.33%)<br><br>**Race/ethnicity:** Not reported<br><br>**Household income:** Not reported	**Housing type:** Not reported<br><br>**Living situation:**<br>• Family of two (SCI/D: 41.38%; household members: 40.00%)<br>• Family of three (SCI/D: 41.38%; household members: 40.00%)<br><br>**Residence characteristics:**<br>• Own their house<br>• People with SCI/D (n = 25, 86.20%)<br>• Household members (n = 26)<br>• Moved after injury<br>• People with SCI/D (n = 15, 51.72%)<br>• Household members (n = 17)<br>• Number of years residing in the house<br>• Mean (People with SCI/D): 9.47 (SD = 8.53)<br>• Range (people with SCI/D): 6	Acceptability<br>Accessibility<br>Adequacy/Accommodation<br>Availability	• Four distinct profiles of priorities were identified: "A Social Participation Hub," "A Family Haven," "A Shared Amenity," & "A Snug Retreat.".<br><br>• Indoor environmental conditions, particularly temperature & air quality, were deemed a major concern among the priorities.<br><br>• Participants across all profiles agreed that the appearance of their home (exterior) should not conveying messages about their identity to others was not a priority.<br><br>• The accessibility related to the immediate surroundings was recognized as a priority in every profile, though its significance varied in the "A Snug Retreat" profile compared to others.<br><br>• Prioritizations of social interactions within the profiles differed. Family members were prioritized in all profiles, with some indicating that they would like to share their space with their family entirely or with family friends, or alone, while others expressed a desire for their home to be shared with many people (e.g., family, friends, guests). Versatile living spaces with various features (e.g., doors) are vital to accommodate varying needs for privacy & social interaction.<br><br>• None of the priorities deemed personal "growth" as a priority.<br><br>• Three out of the four profiles prioritized accessing "immediate surroundings" (e.g., yard, garden).<br><br>• Participants in the "A social participation hub" profile viewed their home as a space that supports a range of

	**Employment status (SCI/D, n = 29):**<br>• Employed (part- or full-time) or student (n = 9, 31.03%)<br>• Retired or at home (n = 20, 68.97%)	months – 32.0 years<br>• Mean (household members: 9.52 (SD = 9.0)<br>• Range (household members): 1.5 - 32.0 years<br><br>**Outcome measures:**<br>• Psycho-environmental housing priorities (PEHP) measure		activities, where mobility inside & outside the home, daily living tasks, domestic responsibilities & work or study are all equally important.
**Labbé et al. [44]**<br>2017<br>Qualitative research – classical content analysis<br>Canada<br>To explore how individuals with disabilities & their family members perceive the home & its features.	**Sample size** (N = 62)**:** SCI/D (n = 31) & their family members (n = 31)<br><br>**Level & severity of injury:**<br>• Paraplegia (n = 14)<br>• Tetraplegia (n = 14)<br>• Not specified (n = 1)<br><br>**Time since injury (in years):**<br>• 7.93 (SD = 2.18)<br><br>**Age (in years):**<br>• SCI/D:<br>• Mean: SCI/D: 44.46 (SD = 14.93) years<br>• Range (SCI/D): 23.1 - 66.9<br>• Median (SCI/D): Not reported<br>• Household members:<br>• Mean (household members): 51.43 (SD = 15.15)<br>• Range (household members): 23.7 - 80.9<br>• Median (household members): Not reported<br><br>**Sex/gender:**<br>• SCI/D<br>• Male (n = 21)<br>• Female (n = 8)<br>• Household members	**Housing type:** Most participants resided in detached or semi-detached houses (owned)<br><br>**Living situation:** All participants with SCI/D (n = 31) lived with their family member(s)<br><br>**Residence characteristics:**<br>• Own their house (SCI/D: n = 25; household members: n = 26)<br>• Moved after injury (SCI/D, n = 15; household members, n = 17)<br>• Mean length of stay in house (SCI/D): 9.47 (SD = 8.53)<br>• Range of stay in dwelling: 6 months – 32 years<br>• Mean length of stay in house (household members): 9.52 (SD = 9.0)<br>• Home modifications:	Acceptability<br>Accessibility<br>Adequacy/Accommodation<br>Availability	• Some levels of modifications were made by all individuals.<br><br>• The bedroom, entire house, living room & kitchen were identified as particularly important for the well-being of individuals with SCI/D & their families.<br><br>• Most participants with SCI/D (77.4%) identified the bedroom as the most important feature whereas family members favored the kitchen, with 83.9% citing it as their preferred space for their well-being.<br><br>• Aids & adaptations for navigating within the home were essential (McNemar [1, n = 62, p = 0.000) & was only mentioned by individuals with SCI/D.<br><br>• The findings indicate that participants had diverse positive experiences with many aspects of their home contributing to well-being, especially when they were able to complete meaningful activities.<br><br>• Individuals with SCI/D (36.7%) & their household members (32.3%) reported the kitchen as an unfavourable aspect. Household members (32.3%) further reported the entire house as unfavourable aspect.<br><br>• More participants with SCI/D reported the basement (36.7%), kitchen (36.7%), as well as indoor/outdoor flooring (30.0%), as unfavourable elements. The

	• Male (n = 8)<br>• Female (n = 22)<br><br>**Race/ethnicity:** Not reported<br><br>**Household income:** Not reported<br><br>**Employment status (SCI/D, n = 31):**<br>• Employed (part- or full-time) or student (n = 9)<br>• Retired or not employed (n = 22)	• Complete adaptation (SCI/D): n = 16<br>• Partial adaptation (SCI/D): n = 7<br>• Introduction for assistive devices without modification to rooms (SCI/D): n = 8<br><br>**Outcome measures:**<br>• Semi-structured interviews<br>• Analytic framework: psycho-environmental potential model		bathroom (33.3%) & immediate surroundings (33.3%) were also reported as unfavourable aspects.<br><br>• Regarding the kitchen, an "open floor plan" was deemed a beneficial element by individuals with SCI/D (n = 20), while the stove was reported as an unfavourable element by several individuals (n = 11). In the bathroom, modifications were reported to contribute to SCI/D participants' well-being (n = 12). The sink & vanity were unfavourable elements reported by participants (n = 10).<br><br>• Individuals with SCI/D highlighted three key reasons that negatively impacted their well-being at home: the functionality of tasks, their physical health & the requirement for support. The primary concern was the difficulty of navigating their living space (McNemar [1, n = 61], p = 0.44).<br><br>• Family members pointed to issues related to instrumentality of tasks & symbolic identification, with their main concern revolving around challenges related to space/size limitations.<br><br>• This study highlighted the need to consider diverse needs of all household members to enhance overall well-being.
**Lijodi et al. [45]**<br>2024<br>Cross-sectional study<br>South Africa<br>To identify the period-prevalence of limitations to activities, restrictions to participation & barriers in the environment in individuals with traumatic SCI/D in Kenya.	**Sample size** (N = 90)**:** Traumatic SCI/D<br><br>**Level & severity of injury:**<br>• Paraplegia (n = 81, 90.00%)<br>Tetraplegia (n = 9, 10.00%)<br>• Complete injury (n = 60, 66.67%)<br>• Incomplete injury (n = 30, 33.33%)<br>• Traumatic injury (N = 90, 100.00%)<br>**Time since injury (in years):**<br>• 1-3 years (n = 35, 38.89%)<br>• 4-6 years (n = 23, 25.56%)<br>• 7-9 years (n = 32, 35.56%)<br><br>**Age (in years):**<br>• Mean: 37.10 (SD = 9.68)<br>• Range: 16 – 60<br>• Median: 34.00	**Housing type:** Not reported<br><br>**Living situation:** Not reported<br><br>**Residence characteristics:** Not reported<br><br>**Outcome measures:**<br>• Sub-modules of the International SCI Community Survey (InSCI) questionnaire<br>• Activity & participation was measured using the following:	Acceptability<br>Accessibility<br>Affordability	• The majority of participants experienced limitations in activities such as toileting (n = 68, 76%), dressing the lower body (n = 57, 63%), dressing the upper body (n = 53, 59%), managing bladder function (n = 50, 56%), & grooming (n = 47, 52%). Participation restrictions included, but were not limited to challenges with using public transportation (n = 81, 90%), standing without support (n = 75, 83%), reaching destinations (n = 68, 76%) & "moving around" (n = 17, 19%).<br><br>• Finances were reported as a barrier by 96% of participants (n = 86), followed by lack of access to public spaces at 92% (n = 83), insufficient equipment for long-distance transportation at 90% (n = 81), issues concerning state services at 87% (n = 78) & access to homes at 76% (n = 68).

	**Sex/gender:**<br>• Male (n = 66, 73.33%)<br>• Female (n = 24, 26.67%)<br><br>**Race/ethnicity:** Not reported<br><br>**Household income (monthly, Kshs):**<br>• <1,100 (n = 36, 40.00%)<br>• 11,001 – 30,000 (n = 29, 32.22%)<br>• 30,001 – 45,000 (n = 17, 18.89%)<br>• 45,001 – 60,000 (n = 6, 6.67%)<br>• 60,001 – 90,000 (n = 1, 1.11%)<br>• 90,001 – 120,000 (n = 1, 1.11%)<br><br>**Employment status:** Not reported	• World Health Organization's (WHO) Model Disability Survey (MDS)<br>• Spinal Cord Independence Measure for Self-Report (SCIM-SR)<br>• Spinal Cord Injury Functional Index (SCI-FI-AT)<br>• Nottwil Environmental Factors Inventory (NEFI)<br>• Telephone administered interviews		• Individuals with tetraplegia were found to be more significantly impacted than those with paraplegia in several areas, including managing daily routines, performing tasks that involve the use of hands & fingers, coping with stress, utilizing private transportation, attending to personal health, completing household chores, engaging with others & maintaining intimate relationships.<br><br>• A lack of adequate communication devices ($p < 0.001$) was identified as a significant barrier identified between individuals with tetraplegia & those with paraplegia.<br><br>• Additionally, significant differences in the period prevalence of environmental barriers concerning the time since injury were found, regarding adverse climatic conditions & inadequate devices for communication. Greater impacts were experienced by individuals who had an SCI/D for a longer period of time.<br><br>• Individuals with tetraplegia faced greater restrictions related to activity & participation.<br><br>• A significant environmental barrier identified in this research was the inadequacy of state services, particularly the lack of disability insurance.
**Manson et al. [46]**<br>2017<br>Qualitative research - Descriptive methodology<br>UK<br>To investigate the experiences of individuals during the days & weeks after they return home following a diagnosis & treatment for metastatic spinal cord compression (MSCC).	**Population studied** (N = 11): MSCC<br><br>**Level & severity of injury:**<br>• Level of MSCC:<br>• C3/4 & L5 (n = 1)<br>• Cx & Lx (n = 1)<br>• L1-3 (n = 1)<br>• L2 (n = 2)<br>• T3 (n = 1)<br>• T3-7 (n = 1)<br>• T5 (n = 1)<br>• T8/9 (n = 1)<br>• T9-12 (n = 1)<br>• T10-11 (n = 1)<br><br>**Time since injury (in years):** Not reported<br><br>**Age (in years):**<br>• Mean: Not reported<br>• Range: 63 – 85	**Housing type:** Not reported<br><br>**Living situation:** Not reported<br><br>**Residence characteristics:** Not reported<br><br>**Outcome measures:**<br>• Semi-structured interviews<br>• Framework analysis techniques were used to analyze interview data.	Acceptability<br>Accessibility<br>Awareness	• Time was identified as a critical factor in participants' descriptions of their MSCC, which included four time points, as stated by the authors: past, present, near future & distant future.<br><br>• When reflecting on the past, participants described various "losses" regarding aspects of their lives, such as their independence, the ability to engage in activities (e.g., going to the store) & manage household tasks.<br><br>• In the present, all participants highlighted the importance of returning home, valuing undisturbed sleep, access to personal belongings & control over daily activities. They faced health challenges due to MSCC (e.g., pain, incontinence, etc.), & leaving the house was difficult due to weakness in the legs or pain. Their mobility was hindered, which made personal care tasks such as washing & dressing difficult. The severity of their symptoms had been less apparent during their

	• Median: Not reported<br><br>**Sex/gender:**<br>• Male (n = 9)<br>• Female (n = 2)<br><br>**Race/ethnicity:**<br>• White British (n = 11)<br><br>**Household income:** Not reported<br><br>**Employment status:** Not reported			hospital stays, revealing new accessibility issues at home (e.g., bed, toilet, furniture, etc.).<br><br>• Some individuals well-supported by available services (e.g., GPs & nurses), while others felt that their needs were not met, leading to frustration. Concerns about services were expressed in both the present & near future time points.<br><br>• In the "Near Future" time point, participants expressed a concern for "getting back to normal," including routines, duties within their home & various activities.
**Maresca et al. [47]**<br>2024<br>Experimental study – employed a parallel, randomized, controlled, non-blinded trial design<br>Italy<br>To evaluate how a home automation system could enhance personal & social independence for individuals with SCI/D, leading to improved cognitive function & lower levels of anxiety & depression.	**Sample size** (N = 50): SCI/D<br><br>**Level & severity of injury:**<br>• AIS-A (n = 24, 48.0%)<br>• AIS-B (n = 26, 52.0%)<br><br>**Time since injury (in years):** Not reported<br><br>**Age (in years):**<br>• Mean: 50.2 (SD = 15.2)<br>• Range: Not reported<br>• Median: Not reported<br><br>**Sex/gender:**<br>• Male (n = 29, 58.0%)<br>• Female (n = 21, 42.0%)<br><br>**Race/ethnicity:** Not reported<br><br>**Household income:** Not reported<br><br>**Employment status:** Not reported	**Housing type:** Not reported<br><br>**Living situation:** Not reported<br><br>**Residence characteristics:** Not reported<br><br>**Outcome measures:**<br>• Montreal Cognitive Assessment (MoCA)<br>• Beck Depression Inventory (BDI)<br>• Hamilton Rating Scale for Anxiety (HRS-A)<br>• Activities of Daily Living (ADL) & Instrumental Activities of Daily Living Scale (IADL)<br>• 12-Item Short-Form Survey (SF-12)<br>• Functional Independence Measure (FIM)<br>• EQ-5D-5L	Acceptability<br>Accessibility<br>Adequacy/Accommodation<br>Availability	• The results indicated significant differences in the effectiveness of the two treatments for ADL & IADL, with improvements noted in both groups. Specifically, ADL scores showed a t-value of 4.97 ($p < 0.001$), while IADL scores had a t-value of 4.64 ($p < 0.001$). Additionally, the experimental treatment groups demonstrated a larger improvement in various areas, including, cognitive skills (measured by MoCA), symptoms of depression (measured by BDI) & activities of daily living (measured by ADL & IADL scale) particularly for the experimental group.<br><br>• Home automation training significantly enhanced activities of daily living, social functioning, quality of life, independence in daily living skills & functional recovery in SCI/D patients, with both traditional & home automation treatments showing positive results.
**Middleton et al. [42]**<br>2022<br>Cross-sectional study<br>Australia<br>To identify prevalent issues across critical	**Sample size** (N = 1,579)**:** SCI/D<br><br>**Level & severity of injury:**<br>• Paraplegia, complete (n = 362, 24.4%)<br>• Paraplegia, incomplete (n = 542, 36.6%)<br>• Tetraplegia, complete (n = 128, 8.6%)	**Housing type:** Not reported<br><br>**Living situation** (n, %):<br>• Living alone: n = 361, 23.0%	Acceptability<br>Accessibility<br>Adequacy/Accommodation<br>Affordability<br>Availability	• While most participants were independent, 73% received daily support.<br><br>• 15-23% of participants reported that accessing private homes or public places posed the biggest environmental challenge. Financial concerns were reported by 16% of

domains of functioning, health, & well-being & to assess self-reported QoL among individuals with SCI/D, by exploring differences based on age, gender, injury characteristics & mobility levels.	• Tetraplegia, incomplete (n = 449, 30.3%)<br>• Traumatic (n = 1,306, 83.6%)<br>• Non-traumatic (n = 257, 16.4%)<br>• Unknown cause (n = 16, 1.0%)<br><br>**Time since injury (in years):**<br>• Mean: 17 (SD = 13.7)<br>• Range: 1 – 72<br><br>**Age (in years):**<br>• Mean: 57.5 (SD = 14.4)<br>• Range: 19 – 94<br>• Median: 59<br><br>**Sex/gender:**<br>• Male (n = 1,157, 73.3%)<br>• Female (n = 422, 26.7%)<br><br>**Race/ethnicity:**<br>• ***Aboriginal or Torres Strait Islander***<br>• No (n = 1,521, 97.6%)<br>• Yes (n = 37, 2.4%)<br><br>**Household income (per annum):**<br>• Less than $23,660 (n = 356, 25.9%)<br>• $23,712 – $35,672 (n = 194, 14.1%)<br>• $35,724 – $47,268 (n = 164, 11.9%)<br>• $47,320 – $62,556 (n = 146, 10.6%)<br>• $62,608 – $80,496 (n = 146, 10.6%)<br>• $80,548 – $100,412 (n = 121, 8.8%)<br>• $100,464 – $123,448 (n = 86, 6.3%)<br>• $123,500 – $154,388 (n = 51, 3.7%)<br>• $154,440 – $206,908 (n = 62, 4.5%)<br>• $206,960 or more (n = 50, 3.6%)<br><br>**Employment status:**<br>• Post-SCI/D employment: 49.9%<br>• 39% of participants between 18 – 65 years (n = 1,078) were employed at time of survey; 50% received disability pension	• Living in an institutional setting: n = 55, 3.5%<br>• Living with other adults: n = 943, 60.1%<br>• Living with kids under 18: n = 72, 4.6%<br>• Living with adults & kids under 18: n = 138, 8.8%<br><br>**Residence characteristics:**<br>• Living place:<br>• Capital city: n = 517, 34.2%<br>• Other metropolitan centre (population > 100,000): n = 347, 23.0%<br>• Large rural centres (population 25,000–99,999): n = 237, 15.7%<br>• Small rural centre (population 10,000–24,999): n = 153, 10.1%<br>• Other rural area (population < 10,000): n = 126, 8.3%<br>• Remote area (population < 5,000): n = 130, 8.6%<br><br>**Outcome measures:**<br>• Aus-InSCI survey questionnaire<br>• Spinal Cord Independence Measure<br>• Modified Spinal Cord Independence Measure-Self Report (m-SCIM-SR)<br>• Nottwil Environmental		participants. Other environmental barriers included issues concerning climate (17%), lack of appropriate transportation for long distances (14%) & inadequate state services (11%).<br><br>• Nearly 96% of participants faced some issues concerning activity or participation. Specifically, 45% struggled with their daily routines & 43% had difficulty completing household duties.<br><br>• Functional independence (assessed by m-SCIM-SR) generally declined with age & was notably lower among recently-injured individuals. Individuals using mobility aids, such as electric or manual wheelchairs, along with those with tetraplegia, frequently faced greater challenges in engaging in activities/participation. Furthermore, males tend to report fewer difficulties in these areas (activity/participation) compared to females.<br><br>• 62% of participants reported their QoL as "good" or "very good," while 13% reported their QoL as "poor" or "very poor." Individuals with non-traumatic SCI/D, whose injury duration was less than 5 years, in "middle range ages," or used mobility aids (electric or assisted manual wheelchairs) reported poorer QoL.

		Factors Inventory Short Form (NEFI-SF)<br>• Spinal Cord Injury – Secondary Conditions Scale<br>• 36-Item Short Form (SF-36) Survey vitality & mental health domains<br>• WHOQOL-BREF quality of life<br>• Injustice Experience Questionnaire<br>• Fatigue Severity Scale<br>• Pittsburgh Sleep Quality Index		
**Noreau et al. [11]**<br>2014<br>Cross-sectional study<br>Canada<br>To determine the most essential service needs of individuals with SCI/D living in the community for at least one year following discharge from rehabilitation, & any assistance they received to address their needs.	**Sample size** (N = 1,549): SCI/D<br><br>**Level & severity of injury:**<br>• Traumatic (n = 1,137)<br>• Non-traumatic (n = 412)<br>• Complete (%, 95% CI): 33.3% (31–36)<br>• Incomplete (%, 95% CI): 66.7% (64-69)<br>• Paraplegia (%, 95% CI): 57.8% (55–60)<br>• Tetraplegia (%, 95% CI): 42.2% (40–45)<br>• AIS<br>◦ AIS-A (%, 95% CI): 36.7% (34–39)<br>◦ AIS-B (%, 95% CI): 7.5% (6–9)<br>◦ AIS-C (%, 95% CI): 19.5% (18–22)<br>◦ AIS-D (%, 95% CI): 20.6% (19–23)<br>◦ AIS-E (%, 95% CI): 2.4% (2–3)<br>◦ Undetermined: 13.3% (12–15)<br><br>**Time since injury (in years):**<br>• Mean: 18.5 (SD = 14.3)<br>• Range: 1 – 81<br><br>**Age (in years):**<br>• Mean: 49.6 (SD = 13.9)<br>• Range: 18 – 90<br>• Median: Not reported<br><br>**Sex/gender (%, 95% CI):**<br>• Male: 67.2% (65–70)<br>• Female: 32.8% (30–35)	**Housing type:** Not reported<br><br>**Living situation (%, 95% CI):**<br>• Single individual living with others: 5.9% (5 – 7)<br>• Single individual living alone: 26.0% (24 – 28)<br>• Living with spouse/partner: 40.8% (38 – 43)<br>• Parent living with spouse or partner & children: 10.1% (9 – 12)<br>• Single parent living with children: 5.2% (4 – 6)<br>• Child living with 2 parents with or without siblings: 8.5% (7 – 10)<br>• Other: 2.5% (2 – 3)	Acceptability<br>Accessibility<br>Adequacy/Accommodation<br>Affordability<br>Availability<br>Awareness	• Since their discharge from rehabilitation, a significant proportion of survey participants reported their most critical needs for community integration, with differences depending on the type of lesion. Participants with traumatic lesions indicated higher expressed needs & met needs compared to those with non-traumatic lesions.<br><br>• 21.4% of participants with a traumatic lesion reported having all their needs met, while this was true for 25.7% of those in the non-traumatic lesion group (Pearson's $\chi 2 = 3.28$, $P = 0.07$).<br><br>• The severity of the lesion significantly impacted the likelihood of individuals with tetraplegia expressing needs related to attendant care, long-distance transportation & accessible housing.<br><br>• Accessible housing needs were expressed by 1,277 participants. 45.2% (42-48, 95% CI) received help from government agencies, 64.6% (62-67, 95% CI) received help from friends & family, 19.2% (17-21, 95% CI) received help from community organizations & 25.3% (23-28, 95% CI) received help from other organizations (e.g., insurance companies, private donations, etc.).<br><br>• Accessible housing was an example of a need that faces considerable barriers overall. The challenges related to the process of acquiring accessible housing, availability of services & physical barriers in the environment, make this need much more difficult compared to other ones. Many individuals (64%) indicated that it was not easy to have this need met.

	**Race/ethnicity (%, 95% CI):**<br>• Majority (White): 91.4% (90–93 CI)<br>• Minorities: 8.6% (7–10 CI)<br><br>**Household income (%, 95% CI):**<br>• <$30,000: 32.1% (30–35)<br>• $30,000 to $59,999: 28.5% (26–31)<br>• $60,000 & over: 39.5% (37–42)<br><br>**Employment status (%, 95 CI):**<br>• Wage/salary: 26.2% (24–28)<br>• Government pension: 27.8% (26–30)<br>• Employer pension: 11.6% (10–13)<br>• Vehicle insurance: 9.7% (8–11)<br>• Provincial program: 11.6% (10–13)<br>• Federal program: 14.8% (13–17)<br>• Do not want to answer: 7.6% (6–9)	• Undeclared: 0.8% (0 – 1)<br><br>**Residence characteristics (%, CI):**<br>• Location of residence (population)<br>• >100,000: 62.2% (58–63)<br>• 10,000-100,000: 16.5% (14–18)<br>• <10,000: 21.3% (19–23)<br><br>**Outcome measures:**<br>• Demographic measure<br>• SCI Health Questionnaire (SCI-HQ)<br>• SCI-related Needs Measure<br>• Participation in Daily Activities Questionnaire (PDAQ)<br>• Quality of life item (1 to 5)<br>• Life Satisfaction-11 (LiSAT-11)<br>• Health care utilization measure (modified version of section about health care utilization from Canadian Community Health Survey (CCHS))<br>• Short Form-12 (SF-12)		
**Norin et al. [48]**<br>2017<br>Cross sectional study<br>Sweden<br>To outline the housing circumstances &	**Sample size** (N = 123): SCI/D<br><br>**Level & severity of injury:**<br>• Tetraplegia AIS A-C (n = 22): 82% traumatic; 18% non-traumatic<br>• Paraplegia AIS A-C (n = 41): 83% traumatic; 17% non-traumatic	**Housing type:**<br>• Tetraplegia AIS A-C: 50% multi-dwelling block; 50% one-family house	Acceptability<br>Accessibility<br>Adequacy/Accommodation<br>Availability<br>Awareness	• More accessibility issues in homes corresponded with reduced participation, reduced autonomy, & increased problems related to participation. Indoor accessibility strongly influenced various aspects of participation: autonomy indoors, family role & participation problems. Accessibility of outdoor surroundings & entrance accessibility were associated with autonomy indoors &

participation issues faced by older adults with long-standing SCI/D, emphasizing SCI/D severity. To investigate the relationship between objective housing accessibility & various participation aspects.	• AIS D (n = 60): 40% traumatic; 60% non-traumatic<br>• Total (N = 123): 62% traumatic; 38% non-traumatic<br><br>**Time since injury (in years):**<br>• Tetraplegia AIS A-C: 30 (SD = 9)<br>• Paraplegia AIS A-C: 27 (SD = 12)<br>• AIS D: 20 (SD = 11)<br>• Total: 24 (SD = 12)<br><br>**Age (in years):**<br>• Mean:<br>• Tetraplegia AIS A-C: 60 (SD = 7)<br>• Paraplegia AIS A-C: 63 (SD = 9)<br>• AIS D: 65 (SD = 9)<br>• Total: 63 (SD = 9)<br>• Range: Not reported<br>• Median: Not reported<br><br>**Sex/gender:**<br>• Male (n = 87, 71%)<br>• Female (n = 36, 29%)<br><br>**Race/ethnicity:** Not reported<br><br>**Household income:** Not reported<br><br>**Employment status:** Not reported	• Paraplegia AIS A-C: 46% multi-dwelling block; 54% one-family house<br>• AIS D: 42% multi-dwelling block; 58% one-family house<br><br>**Living situation:**<br>• Living with partner:<br>• Tetraplegia AIS A-C: 59%<br>• Paraplegia AIS A-C: 51%<br>• All AIS D: 45%<br>• Total: 50%<br><br>**Residence characteristics:**<br>• Housing adaptation<br>o Tetraplegia AIS A-C: 95%<br>o Paraplegia AIS A-C: 90%<br>o All AIS D: 60%<br>o P-value: <0.001 (Tetraplegia AIS A-C vs All AIS D;		family role, but both were not correlated to participation problems.<br><br>• All participants (100%) in the Tetraplegia AIS A-C group used assistance (including, but not limited to, personal assistance, home services, another person, personal security alarms), 66% of the Paraplegia AIS A-C group used assistance & 57% of the All AIS D group used assistance. Overall, 67% of participants used various forms of assistance.<br><br>• The participants reported higher ratings for their participation in activities within the "Autonomy indoors" domain compared to those in the "Family role" domain.<br><br>• Compared to the All AIS D group, individuals in the Paraplegia AIS A-C group indicated better levels of participation in 'Getting around indoors when I want' (P = 0.007).<br><br>• The All AIS D group encountered considerably more barriers in their homes than the Paraplegia AIS A-C group (P < 0.001) & also faced greater indoor obstacles compared to both the Tetraplegia AIS A-C (P < 0.001) & Paraplegia AIS A-C groups (P < 0.001). Additionally, the Tetraplegia AIS A-C group reported more issues concerning both outdoor & indoor areas than the Paraplegia AIS A-C & All AIS D groups.

		Paraplegia AIS A-C vs all AIS D<br><br>**Outcome measures:**<br>• Housing Enabler (HE) tool<br>• Impact on Participation & Autonomy questionnaire (IPA)<br>• Swedish Aging with Spinal Cord Injury Study (SASCIS) data subset		
**Norin et al. [49]**<br>2021<br>Cross-sectional study<br>Sweden<br>To explore housing adaptations & challenges concerning current accessibility experienced by older adults in Sweden who have had an SCI/D for more than 10 years.	**Sample size** (N = 123): SCI/D; one participant excluded (n = 122)<br><br>**Level & severity of injury:**<br>• Tetraplegia AIS A-C (n = 22)<br>• Paraplegia AIS A-C (n = 41)<br>• AIS-D (n = 59)<br><br>**Time since injury (in years):**<br>• Tetraplegia AIS A-C: 30 (SD = 9)<br>• Paraplegia AIS A-C: 27 (SD = 12)<br>• AIS-D: 20 (SD = 11)<br>• Total: 24 (SD = 12)<br><br>**Age (in years):**<br>• Mean:<br>• Tetraplegia AIS A-C: 60 (SD = 7)<br>• Paraplegia AIS A-C: 63 (SD = 9)<br>• AIS-D: 65 (SD = 9)<br>• Total: 63 (SD = 9)<br>• Range: Not reported<br>• Median: Not reported	**Housing type:**<br>• Block of flats (n = 55, 45%)<br>• One-family house (n = 67, 55%)<br><br>**Living situation:**<br>• Living alone (n = 62, 51%)<br>• Cohabiting (n = 60, 49%)<br><br>**Residence characteristics:**<br>• Urban (n = 77, 63%)<br>• Rural (n = 45, 37%)<br>• Housing adaptations<br>• Tetraplegia AIS A-C (n = 21, 100%)<br>• Paraplegia AIS A-C (n = 37, 90%)	Acceptability<br>Accessibility	• Out of the 15 most common housing adaptations, seven were found in the kitchen, while 3 were present at both the entrance & in the bathroom.<br><br>• Housing adaptations were commonly found in entrances (42% of the dwellings), kitchens (31%), & bathrooms (25%).<br><br>• Housing adaptations included: ramps at entrances were reported in 24% of homes, followed by wheelchair-accessible stovetops at 14% & ceiling lifts in bedrooms at 13%.<br><br>• The study revealed that the entrance was the most common site for environmental accessibility barriers, followed by the kitchen & other areas of the home (e.g., bathroom, storage areas, etc.).<br><br>• High wall-mounted cupboards & shelves in the kitchen were the main barrier that led to the most considerable accessibility challenges (accessibility problem score = 10.8).<br><br>• A recurring issue in several areas, including the kitchen (96% of homes), bathroom (86% of homes) & overall indoor spaces (87% of homes), was the presence of controls in inaccessible positions, such as window & door fittings, locks & switches.

	**Sex/gender:**<br>• Male (n = 87, 71%)<br>• Female (n = 35, 29%)<br><br>**Race/ethnicity:** Not reported<br><br>**Household income:** Not reported<br><br>**Employment status:** Not reported	• All AIS D (n = 36, 63%)<br>• Total (n = 94, 79%)<br><br>**Outcome measures:**<br>• Housing Enabler tool<br>• Field notes from observations, photo documentation – detailed descriptions of housing adaptions (including location), perceived assessment challenges & use of mobility devices.<br>• Structured interviews		• Although common housing adaptations like ramps & adjustable cupboards were present in homes, environmental barriers related to level differences, & the inaccessible placement of objects or controls continued to be prevalent in many dwellings.
**Palimaru et al. [50]**<br>2017<br>Qualitative research<br>USA<br>To determine the most significant life aspects for adults with SCI/D & to compare these perspectives between the United States of America (USA) & the United Kingdom (UK).	**Sample size** (N = 20)**:** SCI/D (n_{USA} = 10, n_{UK} = 10)<br><br>**Level & severity of injury:**<br>• Incomplete paraplegia (n_{USA} = 4, 40%; n_{UK} = 3, 30%)<br>• Complete paraplegia (n_{USA} = 2, 20%; n_{UK} = 3, 30%)<br>• Incomplete tetraplegia (n_{USA} = 2, 20%)<br>• Complete tetraplegia (n_{USA} = 2, 20%; n_{UK} = 4, 40%)<br><br>**Time since injury (in years):**<br>• Mean: Not reported<br>• Median: n_{USA} = 8, n_{UK} = 32<br>• Range: n_{USA} = 2-50, n_{UK} = 11-47<br><br>**Age (in years):**<br>• Mean: Not reported<br>• Median: n_{USA} = 57, n_{UK}=51<br>• Range: n_{USA} = 23-69, n_{UK} = 31-62	**Housing type:**<br>• Residential home (n_{USA} = 1, 10%; n_{UK} = 0, 0%)<br><br>**Living situation:**<br>• Alone with no caregiver support (n_{USA} = 2, 20%; n_{UK} = 2, 20%)<br>• With family live-in caregiver support (n_{USA} = 4, 40%; n_{UK} = 4, 40%)<br>• With non-family live in caregiver (n_{USA} = 0, 0%; n_{UK} = 1, 10%)<br>• With someone who is not a caregiver (n_{USA} = 3, 30%; n_{UK} = 3, 30%)	Acceptability<br>Accessibility<br>Adequacy/Accommodation<br>Affordability<br>Availability<br>Awareness	• In the USA group, four participants reported their homes lacked necessary adaptations, such as ramps & accessible bathrooms.<br><br>• All participants in the UK group indicated that their homes were suitably adapted to meet their needs. Examples of adaptations included ramps & wet rooms.<br><br>• A positive financial status was characterized by having sufficient funds to support oneself &/or family (e.g., housing bills), as well as heath & rehabilitation needs (e.g., home modifications, appropriate transportation, etc.).<br><br>• In the USA sample, participants highlighted negative financial status. Also, an individual's health insurance status influences out-of-pocket expenses (e.g., care, rehabilitation, assistive technologies, home modifications). High costs of various products & services were examples of negative financial remarks in the UK sample.

	**Sex/gender:**<br>• Male (n_{USA} = 6, 60%; n_{UK} = 9, 90%)<br>• Female (n_{USA} = 4, 40%; n_{UK} = 1, 10%)<br><br>**Race/ethnicity:**<br>• White (n_{USA} = 8, 80%; n_{UK} = 9, 90%)<br>• Black/African-American (n_{USA} = 1, 10%; n_{UK} = 1, 10%)<br>• Pacific Islander (n_{USA} = 1, 10%; n_{UK} = 0, 0%)<br><br>**Household income:** Not reported<br><br>**Employment status:**<br>• Full-time (30h/week or more) for pay: (n_{USA} = 2, 20%; n_{UK} = 5, 50%)<br>• Part time (less than 30h/week) for pay: (n_{USA} = 1, 10%; n_{UK} = 1, 10%)<br>• Volunteer (no pay): (n_{USA} = 0, 0%; n_{UK} = 1, 10%)<br>• Disabled (not working): (n_{USA} = 3, 30%; n_{UK} = 2, 20%)<br>• Retired: (n_{USA} = 4, 40%; n_{UK} = 1, 10%)	• In a residential home (n_{USA} = 1, 10%; n_{UK} = 0, 0%)<br>• Living with partner at time of interview (n_{USA} = 6, 60%; n_{UK}=6, 60%)<br><br>**Residence characteristics:** Not reported<br><br>**Outcome measures:**<br>• Semi-structured interviews		• Participants (n_{USA} = 4; n_{UK} = 7) indicated that home adaptations positively influenced various domains of QoL, including physical health, mental health & social functioning.<br><br>• The prioritized aspects of QoL shifted right after the injury & also evolved over time as their circumstances changed for some interviewees.<br><br>• All participants emphasized that medical care was a critical factor influencing their QoL.<br><br>• Individuals sought knowledge about SCI/D & life post-injury. Individuals in the USA group reported that they did receive information, such as self-care associated with SCI/D, or wheelchair use, leading them to use the Internet or seek peer support to gain more information.<br><br>• Negative experiences related to care & provision of wheelchairs were reported.<br><br>• Both positive (e.g., wheelchair-accessible vehicles) & negative experiences (e.g., cinemas) regarding accessibility among public infrastructure was shared by participants.
**Pellichero et al. [51]**<br>2023<br>Cross-sectional study<br>France<br>(1) to assess the levels of social involvement among individuals with disabilities residing in Brittany, & (2) to explore their perceptions of both their social & physical surroundings based on their specific disability group.	**Sample size** (N = 25)**:** motor disability (n = 9, 36%; SCI/D, MS), mental health &/or cognitive disability (n = 11, 52%; bipolarity, schizophrenia, autism, Down syndrome), sensory disability (n = 5, 20%; deaf or blind)<br><br>**Level & severity of injury:** Not reported<br><br>**Time since injury (in years):** Not reported<br><br>**Age (in years)**<br>• Mean: 46.0 (SD = 23.6)<br>• Range: Not reported<br>• Median: Not reported<br><br>**Sex/gender:**<br>• Female (n = 10, 40%)<br>• Male (n = 15, 40%)<br><br>**Race/ethnicity:** Not reported<br><br>**Household income:** Not reported	**Housing type:**<br>• Independent housing (n = 17, 68%)<br>• Long-term care facility (n = 6, 24%)<br>• Staying with family or a spouse (n = 2, 8%)<br><br>**Living situation:**<br>• Staying with family or a spouse (n = 2, 8%)<br><br>**Residence characteristics:** Not reported<br><br>**Outcome measures:**<br>• Assessments of Life Habits (LIFE-H, version 4.0)<br>• Measure of the Quality of the	Acceptability<br>Accessibility<br>Adequacy/Accommodation	• In the motor disability group (n = 9), the LIFE-H score was lowest for residence (mean = 4.43, SD = 3.05), while the highest LIFE-H score was for interpersonal relationships (mean = 9.93, SD = 0.17).<br><br>• The LIFE-H subscores revealed that participation scores varied significantly across several areas, influenced by the disability group: daily activities (p = 0.031), mobility (p = 0.006), fitness (p = 0.03) & personal care (p < 0.001).<br><br>• "Support from those around you" was classified as a significant facilitator, whereas "stores & services in your community," "current availability of jobs," "road accessibility," & "time allowed to carry out tasks" were identified as major obstacles among the participants.<br><br>• The MQE scores indicated that perceptions of environmental QoL varied by disability group in several areas, such as 'stores & services' (p = 0.014), 'home care services' (p = 0.006), 'road accessibility' (p = 0.003), 'climatic conditions' (p = 0.025) & 'time allowed to carry out tasks' (p = 0.001).

	**Employment status:**<br>• Employment (n = 7, 28%)<br>• Volunteer activities (n = 10, 40%)<br>• Students (n = 3, 12%)<br>• Retired (n = 5, 20%)	Environment (MQE, version 2.0)<br>• Standardized questionnaires concerning participation & environmental factors<br>• Interviews		• Road accessibility, transportation options & access to stores & services were perceived as significant barriers to mobility. The results also revealed differences in mobility-related participation based on disability type: individuals with cognitive disabilities reported minimal restrictions, while those with motor & sensory disabilities faced substantial limitations. Additionally, participants with motor disabilities reported greater challenges in daily activities, transportation & personal care.
**Pettersson et al. [52]**<br>2015<br>Cross-sectional study<br>Sweden<br>To describe the barriers in the environment related to accessibility & the autonomy of individuals using powered mobility devices (PMD) both indoors & outdoors, to identify significant home-related barriers affecting accessibility in indoor spaces, at entrances, as well as in nearby outside areas & to examine the relationship between personal factors & environmental elements with regard to autonomy in both settings (indoors & outdoors).	**Sample size** (N = 48)**:** SCI/D<br><br>**Level & severity of injury:**<br>• Paraplegia (n = 26)<br>• Tetraplegia (n = 22)<br><br>**Time since injury (in years):**<br>• Mean: Not reported<br>• Range: Not reported<br>• Median: 22<br><br>**Age (in years):**<br>• Mean: Not reported<br>• Range: Not reported<br>• Median: 64<br><br>**Sex/gender:**<br>• Male (n = 33)<br>• Female (n = 15)<br><br>**Race/ethnicity:** Not reported<br><br>**Household income:** Not reported<br><br>**Employment status**: Not reported	**Housing type:** Not reported<br><br>**Living situation:**<br>• Co-habiting (n = 23)<br>• Living alone (n = 25)<br><br>**Residence characteristics:** Not reported<br><br>**Outcome measures:**<br>• Study-specific Swedish Aging with a Spinal Cord Injury Study (SASCIS) questionnaire (for demographic data)<br>• Housing Enabler (HE) tool<br>• Impact on Participation & Autonomy instrument<br>• Canadian Model of Occupational Performance & Engagement	Acceptability<br>Accessibility<br>Adequacy/Accommodation	• 17 participants used their PMDs both indoors & outdoors, whereas 31 participants used their PMDs only outdoors.<br><br>• Almost all PMD users experienced functional limitations related to reduced fine motor skills & poor balance both indoors & outdoors; however, these limitations were significantly less common among those who only used a PMD outdoors only.<br><br>• Environmental barriers caused the most accessibility issues for individuals using PMDs included: mailboxes & trash receptacles that were hard to reach & irregular or uneven surfaces; at entrances, the main obstacles included high thresholds or steps, doors that cannot be secured in the open position & doors that closed quickly or failed to remain open.<br><br>• Participants felt less restricted in their autonomy indoors but experienced more limitations outdoors related to autonomy. A greater number of years living with SCI/D was significantly linked to increased indoor autonomy & less restriction indoors. Additionally, more functional limitations led to increased restrictions in both settings (reduced autonomy indoors & outdoors).<br><br>• There was no significant relationship between the location of PMD use & autonomy, whether in indoor or outdoor settings.<br><br>• In nearly all homes, the controls were found to be positioned too high or in hard-to-reach areas in the kitchen & bathroom. This led the authors to suggest that either home modifications were not carried out by PMD users or they failed to facilitate participation for this population.
**Ronca et al. [53]**<br>2018<br>Cross-sectional study<br>Switzerland	**Sample size**: SCI/D (n = 10,456 identified from hospital discharge data; n = 3,054 identified from SwiSCI data)<br><br>**Level & severity of injury:**	**Housing type:** Not reported<br><br>**Living situation:** Not reported	Accessibility<br>Availability<br>Awareness	• Participants predominantly resided in large cities & in close proximity to specialized SCI/D centres.

(1) To determine which regions people with SCI/D are more likely to reside in compared to the general population, & to assess if particularly vulnerable groups (e.g., elderly individuals or those with tetraplegia) are more likely to live in these areas. (2) To explore whether the residence choice was influenced by the availability of local health care facilities. (3) To identify factors that influence the decision to change residence as individuals age.	• SwiSCI data: 4 people with SCI/D (median) (Q1–Q3, 2–6)/10,000 inhabitants of a medical statistic (Medstat) region<br>• Hospital discharge data: 12 people with SCI/D (median) (Q1–Q3, 8–18)/10,000 inhabitants of a Medstat region<br>• SwiSCI data: 1 person with tetraplegia (median) (Q1–Q3, 0–2)/ 10,000 inhabitants of a Medstat region<br>• Hospital discharge data: 4 people with tetraplegia (median) (Q1–Q3, 3–7)/ 10,000 inhabitants of a Medstat region<br>• SwiSCI data: 1 person aged 65 + (median) (Q1–Q3, 0–2)/ 10,000 inhabitants of a Medstat region<br>• Hospital discharge data: 5 people aged 65 + (median) (Q1–Q3, 3–9)/ 10,000 inhabitants of a Medstat region<br><br>**Time since injury (in years):** Not reported<br><br>**Age (in years):** Not reported<br><br>**Sex/gender:** Not reported<br><br>**Race/ethnicity:** Not reported<br><br>**Household income:** Not reported<br><br>**Employment status:** Not reported	**Residence characteristics:** Not reported<br><br>**Outcome measures:**<br>• Data from nationwide SCI cohort study (SwiSCI) & hospital discharge data was used.		• In 75% of the Medstat regions where participants lived, individuals could reach a specialized SCI/D centre within a 70-minute drive.<br><br>• 60% of SwiSCI patients who completed the aging questionnaire (N = 492) expressed interest moving from their current municipality to a more suitable residential environment that would accommodate their needs as they aged. 28% of participants expressed a consideration to relocate closer to an SCI/D centre as they aged.<br><br>• Although services for healthcare are generally available in Switzerland, this study revealed that this population favours being near to appropriate healthcare facilities.
**Saarimäki et al. [54]**<br>2024<br>Cross-sectional study<br>Finland<br>To explore how often perceived barriers in the environment made participation difficult for the Finnish population with SCI/D & to compare the prevalence of these barriers based on	**Sample size** (N = 1,772)**:** SCI/D [respondents: n = 880; non-respondents: n = 892)<br><br>**Level & severity of injury:**<br>• Respondents<br>◦ Traumatic (n = 490, 56%)<br>◦ Non-traumatic (n = 390, 44%)<br>◦ C1-4 AIS A-C (n = 94, 11%)<br>◦ C5-8 AIS A-C (n = 55, 6%)<br>◦ T1-S5 AIS A-C (n = 184, 21%)<br>◦ AIS D at any injury level (n = 547, 62%)<br>• Non-respondents<br>◦ Traumatic (n = 529, 59%)	**Housing type:** Not reported<br><br>**Living situation (respondents, n = 880):**<br>• With a partner, no children (n = 399, 45%)<br>• Alone, no children (n = 337, 38%)<br>• With a partner & a child/children (n = 96, 11%)	Accessibility<br>Affordability<br>Availability<br>Awareness	• 27.6% of respondents (n = 240) reported that their life was made “a little harder” by inadequate home access, while 18.0% (n = 157) of respondents reported that their life was made “a lot harder.”<br><br>• Challenges related to climate & public accessibility were reported by most participants. Additionally, other significant barriers often identified included transportation for both long & short distances, access to the homes of friends & family, attitudes of friends, political decisions & financial constraints.

gender, age, time since injury & injury severity.	• Non-traumatic (n = 363, 41%)<br>• C1-4 AIS A-C (n = 108, 12%)<br>• C5-8 AIS A-C (n = 62, 7%)<br>• T1-S5 AIS A-C (n = 209, 23%)<br>• AIS D at any injury level (n = 513, 58%)<br><br>**Time since injury (in years):**<br>• Respondents<br>• Mean: 11 (SD = 11)<br>• Range: 1 – 67<br>• Median: 7<br>• Non-respondents<br>• Mean: 10 (SD = 10)<br>• Range: 1 – 66<br>• Median: 6<br><br>**Age (in years):**<br>• Respondents<br>• Mean: 61 (SD = 14)<br>• Range: 20 – 90<br>• Median: 63<br>• Non-respondents<br>• Mean: 54 (SD = 17)<br>• Range: 17 – 93<br>• Median: 55<br><br>**Sex/gender:**<br>• Respondents<br>• Male (n = 574, 65%)<br>• Female (n = 306, 35%)<br>• Non-respondents<br>• Male (n = 638, 72%)<br>• Female (n = 254, 28%)<br><br>**Race/ethnicity:** Not reported<br><br>**Household income:** Not reported<br><br>**Employment status (form of income, respondents, n = 880):**<br>• Work (full-/part-time) (n = 115, 13%)<br>• Sick leave/disability pension/rehabilitation subsidy/unemployed/laid off (n = 352, 40%)	• Other form of residence (n = 45, 5%)<br>• Missing information (n = 3, <1%)<br><br>**Residence characteristics:** Not reported<br><br>**Outcome measures:**<br>• Nottwil Environmental Factors Inventory Short Form (NEFI-SF)<br>• Self-report questionnaire

	• Vocational rehabilitation/student/family leave/other (n = 40, 5%)<br>• Old-age pension/part-time pension (n = 372, 42%)<br>• Missing information (n = 1, <1%)			
**Scovil et al. [55]**<br>2012<br>Observational cohort study<br>Nepal<br>To assess the continuing health & reintegration in the community of individuals with SCI/D following their discharge from inpatient rehabilitation in Nepal.	**Sample size** (N = 37)**:** SCI/D (n = 24), deceased (n = 9)<br><br>**Level & severity of injury (n = 24):**<br>• Complete paraplegia (n = 11)<br>• Incomplete paraplegia (n = 9)<br>• Complete tetraplegia (n = 2)<br>• Incomplete tetraplegia (n = 2)<br><br>**Time since injury (in years) (n = 24):**<br>• Time since injury until admission (mean): 3 years (SD = 4)<br>• Time since injury until admission (range): 2 days to 14 years<br><br>**Age (in years) (n = 24):**<br>• Mean (at admission): 33; SD = 11<br>• Range: 14 – 59<br>• Median: Not reported<br><br>**Sex/gender (n = 24):**<br>• Male (n = 14)<br>• Female (n = 10)<br><br>**Race/ethnicity:** Not reported<br><br>**Household income:** Not reported<br><br>**Employment status:**<br>• Earned enough to be self-sufficient (n = 6)<br>• Earned "some" income (n = 4)<br>• Training (n = 2)<br>• Assisting with household tasks (n = 7)<br>• Unemployed (n = 5)	**Housing type (n = 24):** Not reported<br><br>**Living situation:**<br>• All participants lived with their families, except for two individuals.<br><br>**Residence characteristics:**<br>• Geography: hilly (n = 11); flat (n = 13)<br>• Location:<br>• Rural areas (n = 6)<br>• Village (n = 13)<br>• City (n = 5)<br>• Home ownership<br>• Own (n = 19)<br>• Rent (n = 5)<br>• Community accessibility<br>• Independent access (n = 10)<br>• Assistance required (n = 8)<br>• Inaccessible (n = 6)<br>• House entrance/exit:<br>• Independent access (n = 12)<br>• Assistance required (n = 5)<br>• Inaccessible (n = 7)<br>• Accessible toilet (n = 12)	Acceptability<br>Accessibility<br>Adequacy/Accommodation<br>Affordability<br>Availability	• Accessibility issues at home & in the community were significant barriers for many mobility aid users. Among all wheelchair users, 80% could not independently enter their residences, 74% of those using mobility aids struggled to access the community without help & 6 participants faced challenges with community wheelchair access due to steep terrain, even when assistance was available.<br><br>• Lack of access to an accessible toilet, water source, & road access to homes was found in 1/2 of interviewees & ~66% of wheelchair users. Participants (79% of all participants & 93% of wheelchair users) self-reported these barriers as 'severe' or 'extreme' limitations in participation in the community. Due to these barriers, many individuals relied on neighbours & their family for assistance.<br><br>• Financial worries were reported by 11 participants & a desire for training or employment was expressed by 7 participants.<br><br>• Due to inaccessible environments in the homes, three wheelchair users were unable to their mobility devices to navigate their surroundings.<br><br>• Out of nine participants who made home modifications, eight could only make these modifications with financial help from local non-governmental organizations.<br><br>• Additionally, three wheelchair users moved to urban areas for better accessibility, training & job opportunities.<br><br>• The authors highlighted the importance of considering accessible housing prior to discharge.

		• Access road leading to the house (n = 11)<br>• Accessible water source (n = 11)<br><br>**Outcome measures:**<br>• Modified Barthel Index (MBI)<br>• Participation Scale (P-Scale)<br>• Semi-structured interviews		
**Smith & Caddick [56]**<br>2015<br>Qualitative research<br>UK<br>To investigate the impact of residing in care homes on the health & well-being of individuals with SCI/D.	**Sample size** (N = 20)**:** Traumatic SCI/D & live in a care home<br><br>**Level & severity of injury:**<br>• Traumatic (N = 20)<br><br>**Time since injury (in years):** Not reported<br><br>**Age (in years):**<br>• Mean: 31<br>• Range: 21 – 70<br>• Median: Not reported<br><br>**Sex/gender:**<br>• Male (n = 15)<br>• Female (n = 5)<br><br>**Race/ethnicity:** Not reported<br><br>**Household income:** Not reported<br><br>**Employment status:** Not reported	**Housing type:**<br>• All participants lived in a care home for an average of 2.3 years.<br>• Care home (n = 14)<br>• Living in community, recently left a care home (n = 6)<br><br>**Living situation:** Not reported<br><br>**Residence characteristics:** Not reported<br><br>**Outcome measures:**<br>• Semi-structured interviews	Accessibility<br>Adequacy/Accommodation<br>Awareness	• The QoL of individuals with SCI/D, along with their physical health & psychological well-being, was severely deteriorated by the care home environment.<br><br>• Moving into a care home resulted in a loss of freedom, control over daily activities & the ability to make personal choices, leading to feelings of decreased independence. Participants also reported having to adhere to scheduled meals, recreational activities & care home routines.<br><br>• The environment of the care home restricted individuals' ability to engage in community life, which decreased QoL.<br><br>• Participants encountered difficulties with the re-housing process while in the care home. They reported challenges in obtaining information about re-housing, felt overlooked by society & struggled to identify suitable individuals who could assist them. Some participants were able to contact relevant housing authorities, but they all felt that these authorities overlooked the urgency of their needs & provided inappropriate housing options that were challenging to refuse.<br><br>• Other themes identified include challenges with maintaining relationships, lack of safety in the care home that affected physical health & poor psychological well-being due to restricted participation (e.g., unsuitable physical activities provided for individuals with SCI/D) & loneliness, among others.
**Tate et al. [57]**<br>2023<br>Cross-sectional study<br>USA	**Sample size** (N = 183): SCI/D<br><br>**Level & severity of injury:**<br>• Traumatic (n = 166, 90.70%)<br>• Non-traumatic (n = 17, 9.30%)	**Housing type:** Not reported<br><br>**Living situation:** Not reported	Acceptability<br>Accessibility<br>Affordability<br>Availability	• 25.3%, 13.7% & 24.7% participants, respectively, expressed that their participation was limited by stairs, doors & issues with room temperature. Conversely, ramps & bathroom equipment enabled participation in the home environment for respectively 54.4% & 62.6% of the study participants.

To explore how individual & socio-environmental factors contribute to encouraging healthy aging post-SCI/D.	• AIS-D (n = 46, 25.10%)<br>• Paraplegia, AIS A, B, or C (n = 80, 43.70%)<br>• Tetraplegia, AIS A, B, or C (n = 57, 31.10%)<br><br>**Time since injury (in years):**<br>• Range: 5 – 53<br>• Mean: 23.6 (SD = 11)<br><br>**Age (in years):**<br>• Mean: 59.8 (SD = 8.3)<br>• Range: 45 – 81<br>• Median: Not reported<br><br>**Sex/gender:**<br>• Male (n = 141, 77%)<br>• Female (n = 42, 23%)<br><br>**Race/ethnicity:**<br>• Caucasian (n = 163, 89.00%)<br>• African American (n = 12, 6.60%)<br>• Other (n = 6, 3.30%)<br>• Refused (n = 2, 1.10%)<br><br>**Household income (USD):**<br>• <$25,000: (n = 41, 22.40%)<br>• $25,000 – $39,000 (n = 30, 16.40%)<br>• $40,000 – $59, 000 (n = 29, 15.80%)<br>• $60,000 – $79,999 (n = 27, 14.80%)<br>• ≥ $80,000 (n = 45, 24.60%)<br>• Refused (n = 11, 6.00%)<br><br>**Employment status:**<br>• Not employed (n = 133, 72.70%)<br>• Employed part-time (n = 20, 10.90%)<br>• Employed full-time (n = 29, 15.80%)<br>• Refused (n = 1, 0.50%)	**Residence characteristics:**<br>• Living in urban areas (n = 151, 82.50%)<br>• Living in rural areas (n = 28, 15.30%)<br>• Unknown (n = 4, 2.20%)<br><br>**Outcome measures:**<br>• Comorbidities Questionnaire (this questionnaire is according to a list of conditions outlined by the Centers for Medicare & Medicaid Services (CMS).<br>• SCI Secondary Conditions Scale (SCI-SCS)<br>• Spinal Cord Injury Functional Index/Assisted Technology version (SCI-FI/AT)<br>• Behavioral Risk Factor Surveillance System (BRFSS)<br>• SCI-QOL Satisfaction with Social Roles & Activities scale – part of the Spinal Cord Injury Quality of Life<br>• Cohen's Social Network – Social Integration Index<br>• Formal Social Integration scale used in analyses<br>• MOS Emotional/Information Support scale – part of the MOS Social Support Survey	• 54.9%, 58.8% & 63.7% of participants respectively expressed how curb cuts, elevators & paved surfaces aided in their participation within the community-built environment.<br><br>• Improved access to features in the home environment & appliances was linked to lower scores in secondary conditions (P = 0.002).<br><br>• Individuals who did not provide family income data exhibited significantly lower SCI-SCS scores compared to individuals with incomes under $25,000 USD (P = 0.043).<br><br>• The findings highlight the importance of the built home environment, satisfaction related to activities & social roles, & resource accessibility as predictors of secondary conditions.<br><br>• Individuals with private health insurance (not including Auto No Fault) exhibited lower SCI-SCS scores compared to those covered by Auto No Fault or Workers Compensation (P = 0.010).<br><br>• Environmental factors & health insurance should be carefully considered to reduce the risk of developing secondary conditions among individuals aging with SCI/D.

		• Facilitator & Barriers Survey for Mobility (FABS-M)		
**Angulo et al. [58]**<br>2019<br>Cross-sectional study<br>Spain<br>Grey literature<br>To examine the QoL of individuals with SCI/D in the third stage of this condition, when they are engaged in daily social interactions. The period in which individuals with SCI/D continue with their daily life outside of the hospital is referred to as the *third stage* [69].	**Sample size** (N = 105): SCI/D<br><br>**Level & severity of injury:**<br>• Paraparesis (n = 12; 11.4%)<br>• Paraplegia (n = 56; 53.3%)<br>• Quadriparesis (n = 7; 6.7%)<br>• Quadriplegia (n = 26; 24.8%)<br><br>**Time since injury (in years):**<br>• Mean: 16.54 (SD = 12.61)<br>• Range: 1 – 66<br><br>**Age (in years):**<br>• Mean: 42.36 (SD = 11.02)<br>• Range: 19 – 73<br>• Median: Not reported<br><br>**Sex/gender:**<br>• Male (n = 73; 69.5%)<br>• Female (n = 32; 30.5%)<br><br>**Race/ethnicity:** Not reported<br><br>**Household income (monthly, EUR):**<br>• < 1000 € (n = 57, 54.3%)<br>• 1000-3000 € (n = 28, 26.7%)<br>• >3000 € (n = 12, 11.4%)<br><br>**Employment status:** Not reported	**Housing type:** Not reported<br><br>**Living situation:** Not reported<br><br>**Residence characteristics:**<br>• Adapted housing (n = 86, 81.9%)<br>• Non-adapted housing n = 19, 18.1%)<br><br>**Outcome measures:**<br>• Quality of Life Questionnaire (QoLQ)<br>• Daily Stress Scale for People with SCI/D<br>• Somatic Symptoms Scale for People with SCI/D<br>• Stress-Hardiness Personality Questionnaire<br>• Social Support Behaviors (SS-B) Scale<br>• Use of medication – this was used to gain insight into 'objective indicators' of SCI/D symptoms	Acceptability<br>Accessibility<br>Adequacy/Accommodation<br>Availability	• Many individuals with SCI/D had their homes adapted (81.9%), while some had not (18.1%), indicating challenges related to the cost, time, or relocation to a residence with home modifications. Higher self-perceived QoL was reported in individuals with adapted housing. This positive association was reflected in QoLQ scores (p<0.01).<br><br>• The household income of 54.3% of the families that participated in the study had a monthly income of lower than 1000 €, while 26.7% had a monthly income between 1000 € & 3000 €. 11.4% of the participants had a monthly household income of over 3000 €. Authors reported that this aligns with high rates of unemployment observed in SCI/D populations.<br><br>• The amount of time post-SCI/D allows individuals to adapt to their environment & their lifestyle.
**Lister et al. [59]**<br>2024<br>Observational cross sectional descriptive design<br>South Africa<br>Grey literature<br>The study aimed to: (i) create a demographic profile	**Sample size** (N = 60):<br>• SCI/D & spasms (n = 28, 47%)<br>• Cerebral palsy (n = 5, 8%)<br>• Congenital deformity (n = 6, 10%)<br>• Frail & fracture (n = 3, 5%)<br>• Amputation (n = 8, 13%)<br>• Poliomyelitis (n = 1, 2%)<br><br>**Level & severity of injury:** Not reported	**Housing type:** Not reported<br><br>**Living situation:** Not reported<br><br>**Residence characteristics:** Not reported<br><br>**Outcome measures:**	Acceptability<br>Accessibility<br>Adequacy/Accommodation	• Two participants (3%) had access to an indoor accessible toilet, five participants (8%) had access to an outdoor accessible toilet & 26 participants (43%) had a pit privy.<br><br>• 26 (43%) of participants had a standard indoor toilet, while 1 participant (2%) had a standard outdoor toilet.<br><br>• Inside the home, participants reported that the most common activities included watching television (n =3 8), cooking (n = 24), eating (n = 13), doing laundry (n = 9),

of standard wheelchair users in Thulamela Municipality, Vhembe District; (ii) evaluate the importance & satisfaction among wheelchair users with regard to completing activities of daily living & instrumental activities of daily living, both at home & outside; & (iii) assess their satisfaction, comfort & body position while using prescribed standard wheelchairs.	**Time since injury (in years):** Not reported<br>**Age (in years):**<br>• Mean: 45.08 (SD = 19.07)<br>• Range: 18 – 93<br>• Median: 39.50<br><br>**Sex/gender:**<br>• Male (n = 34, 57%)<br>• Female (n = 26, 43%)<br><br>**Race/ethnicity:** Not reported<br><br>**Household income:** Not reported<br><br>**Employment status:** Not reported	• Wheelchair Outcome Measure (WhOM)	cleaning (n = 8), bathing (n = 5) & moving around the yard (n = 5). In contrast, the least common activities included looking after children (n = 1), studying (n = 2), playing games (n = 2), going to the bathroom (n = 2) & gardening (n = 3).<br><br>• The level of importance of participation in activities in the home was rated as high with a mean score of 9.36 (SD = 1.46) among wheelchair users, while their satisfaction in these activities averaged at 6.93 (SD = 3.18) on a scale of 0 to 10.<br><br>• Outside the home, most common activities included visiting friends & family (n = 14), with a high level of importance (mean: 9.86; SD = 0.53) & satisfaction (mean: 8.43; SD = 2.28). Other common activities included going shopping (n = 11) & going to the clinic (n = 11).<br><br>• The level of importance of activity participation exterior to the home was rated as high with a mean score of 9.28 (SD = 1.38) among wheelchair users, while their satisfaction in these activities averaged at 6.72 (SD = 3.11) on a scale of 0 to 10.<br><br>• The study revealed that participants placed a high level of importance on activities at home, but their satisfaction with their ability to do so using a wheelchair was limited. Despite the high level of importance they placed on these activities, participants were not satisfied with how well wheelchair use facilitated their participation.
